# Met–HER3 crosstalk supports proliferation via MPZL3 in *MET*-amplified cancer cells

**DOI:** 10.1007/s00018-022-04149-w

**Published:** 2022-03-05

**Authors:** Yaakov E. Stern, Abdulhameed Al-Ghabkari, Anie Monast, Benoit Fiset, Farzaneh Aboualizadeh, Zhong Yao, Igor Stagljar, Logan A. Walsh, Stephanie Duhamel, Morag Park

**Affiliations:** 1 Rosalind and Morris Goodman Cancer Institute, McGill University, Montréal, QC Canada; 2 Department of Biochemistry, McGill University, Montréal, QC Canada; 3 Donnelly Centre, University of Toronto, Toronto, ON Canada; 4 Department of Biochemistry, University of Toronto, Toronto, ON Canada; 5 Department of Molecular Genetics, University of Toronto, Toronto, ON Canada; 6 Mediterranean Institute for Life Sciences, Meštrovićevo Šetalište 45, 21000 Split, Croatia; 7 Department of Human Genetics, McGill University, Montréal, QC Canada; 8 Department of Medicine, McGill University, Montréal, QC Canada; 9 Department of Oncology, McGill University, Montréal, QC Canada

**Keywords:** Crosstalk, MPZL3, HER3, MET, Tyrosine, Signaling

## Abstract

Receptor tyrosine kinases (RTKs) are recognized as targets of precision medicine in human cancer upon their gene amplification or constitutive activation, resulting in increased downstream signal complexity including heterotypic crosstalk with other RTKs. The Met RTK exhibits such reciprocal crosstalk with several members of the human EGFR (HER) family of RTKs when amplified in cancer cells. We show that Met signaling converges on HER3–tyrosine phosphorylation across a panel of seven *MET*-amplified cancer cell lines and that HER3 is required for cancer cell expansion and oncogenic capacity in vitro and in vivo. Gene expression analysis of HER3-depleted cells identified MPZL3, encoding a single-pass transmembrane protein, as HER3-dependent effector in multiple *MET*-amplified cancer cell lines. MPZL3 interacts with HER3 and MPZL3 loss phenocopies HER3 loss in *MET*-amplified cells, while MPZL3 overexpression can partially rescue proliferation upon HER3 depletion. Together, these data support an oncogenic role for a HER3–MPZL3 axis in *MET*-amplified cancers.

**Supplementary Information:**

The online version contains supplementary material available at 10.1007/s00018-022-04149-w.

## Introduction

Receptor tyrosine kinases (RTKs) have been among the earliest and most effective targets for precision medicine in human cancer [1]. RTK activation canonically proceeds through receptor homo-oligomerization and *trans*-auto-phosphorylation, but many cases of heterotypic signaling between different RTKs, frequently referred to as crosstalk, have been reported in the literature [2, 3]. Epidermal growth factor receptor (EGFR)-family RTKs in particular have become targets for clinical intervention in human cancer. The EGFR family contains four paralogous receptor tyrosine kinases that evolved from a single-precursor EGFR homologue and exhibits extensive crosstalk with each other [1]. EGFR, human epidermal growth factor receptor 2 (HER2) and HER3 are frequently overexpressed in human cancers and have been shown to induce canonical cancer-associated signals upon their activation by mutation, gene amplification or constitutive ligand presentation [1, 4]. EGFR and HER2 have been successfully targeted both by ATP-competitive small molecule inhibitors as well as by antibody-based therapies that bind to the extracellular domains of these RTKs. These interventions are part of the standard of care in lung, breast, and colorectal cancer therapy [5, 6].

Unlike EGFR and HER2, their paralogue HER3 does not possess intrinsic kinase activity due to substitutions at critical positions in the kinase domain [7]. To phosphorylate tyrosine residues in the HER3 cytoplasmic tail to recruit and activate intracellular signaling molecules, it must interact with another functional tyrosine kinase [8]. HER3 exhibits this crosstalk preferentially with HER2 and EGFR, with which it can form heterodimers [1, 9]. Ligand-induced hetero-dimerization with EGFR or HER2 promotes HER3–tyrosine phosphorylation at 6 positions (1054, 1197, 1222, 1260, 1276, and 1289) in the C-terminal tail capable of recruiting of the SH2-domain-containing p85 activator of phosphatidyl-inositol-3 kinase (PI3K) [10, 11] and promoting activation of the downstream Akt signaling pathway [10, 12]. Such activation of HER3 signaling by EGFR and HER2 is observed in lung and breast cancers, and combinatorial targeting of HER3 along with EGFR and HER2 via antibodies has been validated as a means to increase the efficacy of therapeutic intervention and delay or prevent the onset of acquired resistance [13–15].

The Met RTK, which has been assessed as a therapeutic target in multiple human solid tumors, has also been shown to engage in crosstalk with EGFR-family RTKs, including HER3 [reviewed in 16–19]. *MET*-amplified cancer cell lines are exquisitely dependent on Met signaling for proliferation and survival through the activation of downstream mitogen-activated protein kinase (MAPK), PI3K-Akt and STAT3 signaling pathways [20, 21]. Crosstalk between Met and EGFR family RTKs is observed in many cancer cell lines derived from *MET*-amplified lung, gastric, esophageal and other cancers [recently reviewed in 2]. This has led to efforts to clinically target crosstalk between Met and EGFR itself, but despite combinatorial inhibition of Met and EGFR showing improved efficacy in cell culture and xenograft mouse models of non-small-cell lung cancer with *MET* amplification and *EGFR* mutations [22–25], this benefit was not supported in clinical trials [23]. Nonetheless, *MET* amplification is recognized as a mechanism of resistance following small-molecule inhibitor targeting of EGFR mutant lung cancer, and conversely, EGFR and HER3 amplification or mutation have been observed as resistance mechanisms to Met inhibition in human cancers [26–28]. In addition, ligand-mediated activation of EGFR-family RTKs, including HER3, can restore cell proliferation in *MET-*amplified cells treated with a small-molecule Met RTK inhibitor [29]. Thus, it remains critical to understand RTK crosstalk and its contribution to tumorigenesis as well as its role in the emergence of acquired resistance to RTK inhibitors in many cancer patients. Further elucidation of the mechanisms underlying RTK crosstalk downstream of Met will be important to improve patient stratification for the use of Met inhibitors and to effectively exploit co-vulnerabilities in Met and EGFR family signaling.

Here, we investigate crosstalk between Met and the EGFR family of RTKs using a panel of *MET*-amplified cancer cell lines as a model of Met-dependent cancers. We report selection of a Met–HER3 signaling axis across a panel of *MET*-amplified cancer cell lines that is required for cell proliferation. We identify a novel interactor of HER3, MPZL3, that is recruited in a Met-dependent manner and is required for efficient proliferation downstream of HER3 in *MET*-amplified cell lines. We further suggest that the HER3–MPZL3 axis may contribute broadly to oncogenic activity in RTK-dependent human cancers.

## Results

### *Met crosstalk with the EGFR family converges on HER3 in *MET*-amplified cells*

**Fig. 1:**
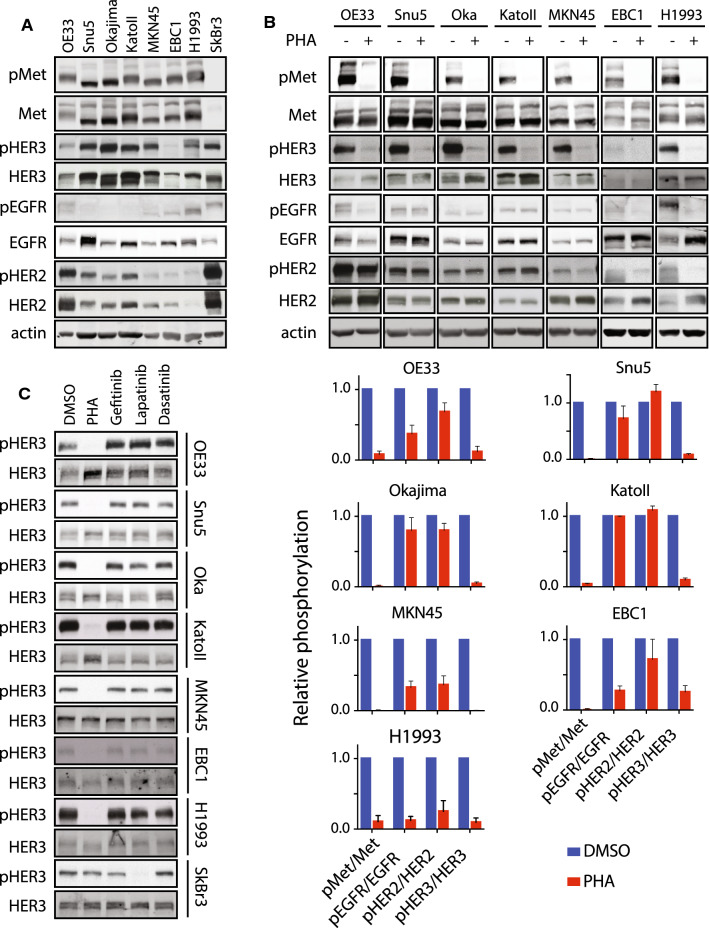
*MET*-amplified cancer cells display Met–EGFR family crosstalk with preference for HER3. **a** Western blot analysis of the phosphorylation of Met (Tyr^1234/45^), EGFR (Tyr^1173^), HER2 (Tyr^1221/22^) and HER3 (Tyr^1289^) in seven *MET*-amplified cancer cell lines and the *ERBB2*-amplified cell line SkBr3. **b** Western blot analysis of the phosphorylation of Met (Tyr^1234/45^), EGFR (Tyr^1173^), HER2 (Tyr^1221/22^) and HER3 (Tyr^1289^) in the indicated cell lines treated with PHA-665752 (PHA) (0.5 μM, 1 h) or DMSO. Histogram shows quantification of phosphorylated RTK in treated vs. control conditions, normalized to total protein (lower panel, *n* = 3). **c** Western blot analysis of the phosphorylation of HER3 (Tyr^1289^) in the indicated cell lines treated with PHA (0.5 μM); the EGFR inhibitor, gefitinib (1 μM); the HER2/EGFR inhibitor lapatinib (1 μM); or the Src and Abl family kinase inhibitor, dasatinib (0.1 μM), for 1 h (*n* = 3). Error bars indicate SEM

To understand the crosstalk signaling axis between Met and EGFR family RTKs, we assembled a panel of *MET*-amplified cancer cell lines, derived from esophageal (OE33), gastric (KatoII, Okajima, Snu5, MKN45) and lung (EBC1, H1993) adenocarcinomas. SkBr3 cells were included as a control, in which EGFR, HER2 and HER3 phosphorylation is driven by *ERBB2* amplification and dependent on HER2 kinase activity [30] (Fig. 1c and Supplementary Fig. 1b). High levels of constitutively tyrosine phosphorylated Met protein were observed in OE33, KatoII, Okajima, Snu5, MKN45, EBC1, and H1993 cells as previously reported [21, 31, 32], while SkBr3 cells did not express Met at a detectable level (Fig. 1a). We assessed a protein levels and tyrosine phosphorylation of EGFR family members EGFR, HER2 and HER3 by Western blot. Levels of the three EGFR-family receptors vary across the seven MET-amplified cell lines tested, and although all three receptors are expressed, they are tyrosine phosphorylated to different levels (Fig. 1a). To establish whether EGFR, HER2 or HER3 phosphorylation is dependent on Met activity in our panel, we treated the panel of cell lines for one hour with PHA-665752 (PHA) a small-molecule tyrosine kinase inhibitor highly selective for Met over EGFR and HER2 at sub-micromolar concentrations, at 0.5 µM [33, 34]. While EGFR and HER2 were basally tyrosine phosphorylated in all cell lines tested, tyrosine phosphorylation of EGFR was dependent on Met kinase activity in three out of seven tested cell lines (OE33, EBC1 and H1993) and tyrosine phosphorylation of HER2 was dependent on Met activity in two lung cancer cell lines tested (EBC1 and H1993) (Fig. 1b). Tyrosine phosphorylation of HER3, by contrast to EGFR and HER2, was dependent on Met activity in all seven *MET*-amplified cell lines tested (Fig. 1b). This supports a preference for HER3 among EGFR family RTKs for Met-dependent tyrosine phosphorylation.

As the HER3 kinase domain possesses impaired intrinsic tyrosine kinase activity, tyrosine phosphorylation of HER3 is normally attributed to its hetero-dimerization with EGFR or HER2 upon binding its ligand neuregulin, thus utilizing EGFR or HER2 kinase activity to induce HER3-dependent signaling [1]. While tyrosine phosphorylation of EGFR and HER2 remained mostly independent of Met activity in our panel of *MET*-amplified cell lines, it was possible that Met-dependent HER3–tyrosine phosphorylation also proceeded through an EGFR- or HER2-mediated mechanism. Additionally, Src family non-receptor tyrosine kinases are recruited to HER3 [35]. Src overexpression has been shown to promote HER2-HER3 association [36], suggesting that Src could contribute to HER3 phosphorylation downstream of Met either directly or via HER2. Furthermore, Src can phosphorylate EGFR in the kinase activation loop, which potentiates EGFR kinase activity and signal output, indicating that Src could indirectly contribute to HER3 phosphorylation via an EGFR-dependent mechanism [37].

To test whether HER3 phosphorylation proceeded through one of these mechanisms in *MET*-amplified cells, we treated the Met-dependent cell lines in our panel with PHA (0.5 µM); the EGFR small molecule kinase inhibitor, gefitinib (1 µM); the HER2 small molecule kinase inhibitor, lapatinib (1 µM); the Src family broad-spectrum kinase inhibitor dasatinib (0.1 µM); or DMSO as control. Tyrosine phosphorylation of HER3 in all cell lines studied was decreased following inhibition of the Met kinase using PHA but not with any of the other inhibitors tested, indicating that Met kinase activity is primarily responsible for HER3–tyrosine phosphorylation in these MET-amplified cells (Fig. 1c). We expand these observations using additional Met inhibitors Crizotinib (0.1 µM) and Tepotinib (0.1 µM), currently used in the clinic with patients with advanced solid tumor [38, 39] by studying phosphorylation of additional HER3–tyrosine residues (Y1222 and Y1289), known to participate in phosphatidylinositol-3 kinase signaling [12]. These results demonstrate that all three Met RTK inhibitors efficiently decrease HER3 phosphorylation on both tyrosine residues (Supplementary Fig. 1a). In addition, we found that HER2 phosphorylation was sensitive to lapatinib but not PHA in Snu5, Okajima, KatoII and OE33 cells, indicating that the Met–HER3 signaling axis is independent from HER2 activity in these *MET*-amplified cancer cells. Furthermore, we found that phosphorylation of EGFR was sensitive to gefitinib only in control PC9 cells which harbor an oncogenic exon-skipping mutation in the *EGFR* gene and was dependent on either Met or HER2 in cells with *MET* or *ERBB2* amplification, as previously reported [21, 26, 40] (Supplementary Figs. 1b, c). Together, these data demonstrate that HER3–tyrosine phosphorylation is primarily regulated by Met in a *MET*-amplified setting, and the consistent observation of this across seven independently derived cell lines further indicates that tyrosine phosphorylation of HER3 by Met is under strong selection in *MET*-amplified cancers.

### HER3 depletion impairs proliferation and reduces tumor growth in Met-dependent cancer cells

**Fig. 2:**
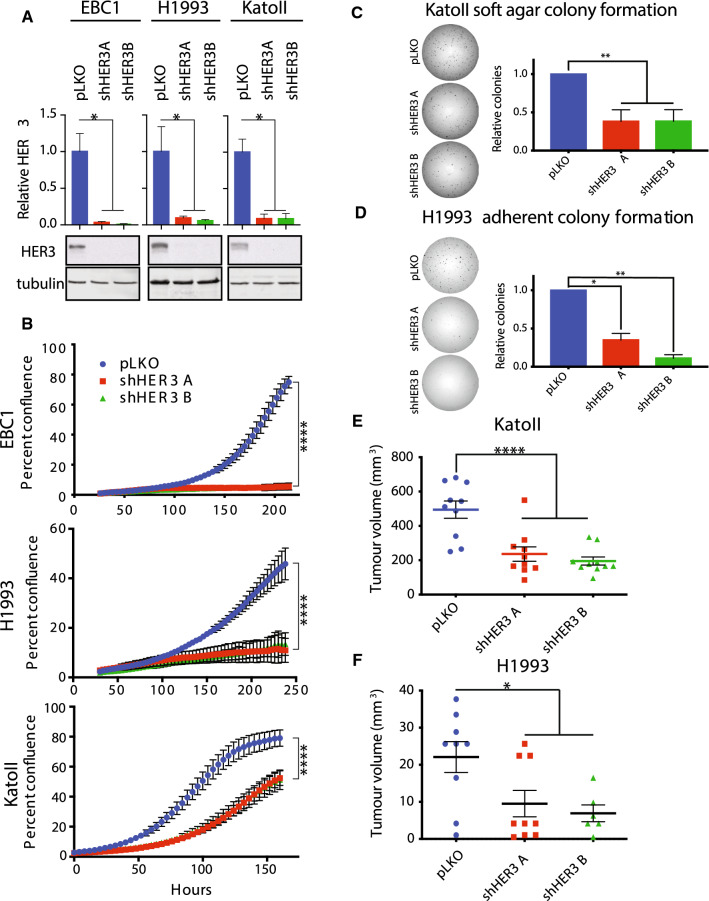
HER3 is required for expansion of *MET*-amplified cancer cells in vitro and in vivo. **a** Western blot analysis of total and phosphorylated levels of HER3 in the indicated cell lines stably transduced with control (pLKO) or shRNAs targeting HER3. Quantification of HER3 relative to tubulin (*n* = 3). **b** Live-cell imaging to measure cell confluence of the indicated cell lines stably transduced with control (pLKO) or shRNAs targeting HER3. Representative replicates are shown (*n* = 3); error bars indicate SD. **c**–**d** Representative images of colony-forming assay are presented. **c** Quantification of colony-forming capacity of KatoII cells in soft agar (colonies > 200 μm) (*n* = 4). Error bars indicate SEM. **d** Quantification of colony-forming capacity in H1993 cells (*n* = 3). Error bars indicate SEM. **e**–**f** Tumor growth of the indicated cell lines engraft in the flank of immunocompromised mice (NSG), *n* = 10. One-way ANOVA with Dunnett’s test for multiple comparisons: *p ≤ 0.05; **p ≤ 0.01; ***p ≤ 0.001; ****p ≤ 0.000

Recurrence of a Met–HER3 crosstalk axis across multiple *MET*-amplified cancer cell lines further suggests that HER3 may act as a ubiquitous signal transducer downstream from Met in this context. To test whether HER3 contributed to the oncogenic transformation in *MET*-amplified cancer cells, we depleted HER3 using two independent shRNA hairpins in *MET-*amplified EBC1, H1993, and KatoII cells (Fig. 2a). HER3 depletion decreased cell proliferation in all three *MET*-amplified cell lines tested when compared to controls (Fig. 2b). HER3 knockdown also impaired the ability of KatoII cells to form colonies in soft agar, indicating that impaired cell expansion also affects oncogenic phenotypes canonically associated with Met-dependent transformation (Fig. 2c) [21]. In H1993 cells, which do not form colonies in soft agar under normal conditions (data not shown), HER3 knockdown impaired colony-forming capacity in 2D cell culture (Fig. 2d). This stood in contrast to our observations in non-*MET-*amplified HeLa cells, in which HER3 knockdown did not exert any effect on clonogenic capacity in 2D cell culture (Supplementary Fig. 2a). This led us to test whether HER3 was required for *MET*-amplified tumor formation in vivo.

To evaluate if HER3 depletion impacted tumorigenicity, we subcutaneously injected immune-deficient NSG mice with H1993 and KatoII cells harboring stable knockdown of HER3, or control cells infected with an empty shRNA expression vector (pLKO). Tumors formed rapidly from KatoII control cells, whereas tumors grown from HER3-depleted cells formed with delayed kinetics and were smaller than control tumors at all time points (Fig. 2d and Supplementary Fig. 2b). Tumors from HER3-depleted cells did not re-express HER3 protein 18 days following the injection, demonstrating that knockdown remained stable through the length of the experiment. However, HER3 depletion was insufficient to completely abrogate tumor growth (Supplementary Fig. 2c). Subcutaneous tumors from H1993 control cells similarly grew more rapidly than those grown from HER3-depleted cells (Fig. 2f). Our results demonstrate that knockdown of HER3 significantly impairs the outgrowth of *MET*-amplified xenograft tumors in vivo.

### Gene expression analysis of HER3-dependent transcripts across MET-amplified cell lines

**Fig. 3:**
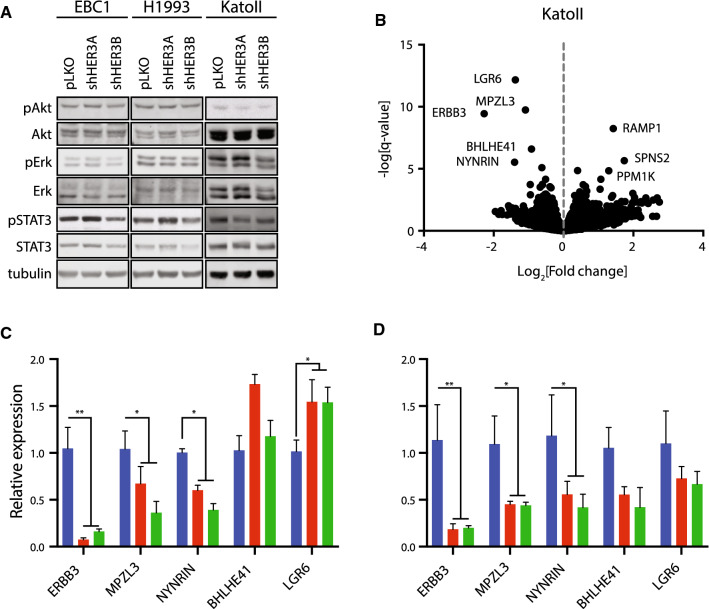
Gene expression analysis identifies *MPZL3* as a HER3-regulated transcript in *MET*-amplified cells. **a** Western blot analysis of the phosphorylation of Akt (Ser^473^), ERK1/2 (Thr^202^/Tyr^204^) and STAT3 (Tyr^705^) in the indicated cell lines stably transduced with control (pLKO) or shRNAs targeting HER3. **b** Volcano plot of RNA sequencing data. Genes with significant increase or decrease in expression between control (pLKO) and HER3-depleted KatoII cell lines are labeled. **c**–**d** Measurement of the relative amounts of *ERBB3*,* MPZL3*,* NYNRIN*,* BHLHE41* and *LGR6* genes in the indicated cell lines by RT-qPCR. **c** Genes downregulated under steady-state conditions upon stable HER3 depletion in EBC1 cells. **d** Genes downregulated under steady-state conditions upon stable HER3 depletion in H1993 cells. Data are means (bars) of three individual replicates. Two-way ANOVA with Dunnett’s test for multiple comparisons; *p ≤ 0.05; **p ≤ 0.01; ***p ≤ 0.001; ****p ≤ 0.0001. Error bars indicate SEM

We hypothesized that depletion of HER3 would affect proliferation in *MET*-amplified cells by reducing the phosphorylation of signaling pathway proteins canonically activated downstream of Met and HER3. To test this, we analyzed protein extracts from HER3-depleted EBC1, H1993 and KatoII cells for phosphorylation of the activation loop residues (T202/Y204) on ERK1/2 for MAPK pathway activity and markers of mTORC2 activity (S473) on the Akt signaling transduction protein to test PI3K pathway activation. Phosphorylation of these molecules and the activation of their downstream transcriptional targets, along with tyrosine phosphorylation of the dimerization domain at position 705 in the transcription factor STAT3, have been shown to be involved in proliferation, colony formation and tumorigenesis in a panel of *MET*-amplified gastric cancer cell lines, including KatoII [21]. However, under these conditions we did not observe significant changes in these signaling pathways upon HER3 depletion by two different shRNAs (Fig. 3a) nor did we did not observe a significant increase in apoptosis by annexin-V positivity (Supplementary Fig. 3). Together, these data support a phenotype of HER3 depletion in *MET*-amplified cells that is not dependent on the inhibition of canonical HER3-associated signaling pathways, but on potential as-yet uncharacterized functions of HER3.

To identify HER3-regulated genes in *MET*-amplified cells, we performed gene expression profiling by RNA sequencing of KatoII cells depleted of HER3 by two different shRNAs to identify significantly altered transcripts (genes with False Discovery Rate (FDR) < 0.05, and log2 fold change ≥ 1.0 were considered). Genes with a decrease in abundance upon HER3 knockdown included *ERBB3* as well as *LGR6*, *MPZL3* and *NYNRIN*, while genes elevated in expression (log2 FC ≥ 1, FDR < 0.05) included *PPM1K*, *RAMP1*, and *SPNS2* (Fig. 3b). We expanded our analysis to include BHLHE41, a transcription factor involved in mammalian circadian regulation and a repressor of differentiation in skeletal muscle and the immune system [41]. BHLHE41 was significant in our analysis with a HER3-dependent fold change just below our cutoff. To validate which transcripts were recurrently dependent on HER3 in a *MET*-amplified setting, we measured the expression of genes decreased in KatoII cells by quantitative RT-PCR in *MET*-amplified EBC1 and H1993 cells following HER3 knockdown.

Of the genes surveyed, *ERBB3*, *MPZL3,* and *NYNRIN* consistently demonstrated a decrease in mRNA levels in all HER3-depleted cells tested (EBC1, KatoII and H1993) (Figs. 3c and d). *LGR6* was unaffected and *BHLHE41* was decreased in expression in H1993 and KatoII but not in EBC1 cells. None of the genes elevated in expression upon HER3 knockdown in KatoII cells were differentially expressed in EBC1 or H1993 cells (Supplementary Fig. 4). *NYNRIN* is a gene with a putative RNA-binding function, but it has not been characterized biochemically to our knowledge [42]. *MPZL3* is a predicted adhesion receptor with a role in epidermal differentiation but no previously characterized role downstream of RTKs [43, 44]. Therefore, we proceeded to delineate the role of MPZL3 in Met–HER3 crosstalk in *MET*-amplified cells.

### MPZL3 is required for proliferation downstream of HER3 in MET-amplified cells

**Fig. 4:**
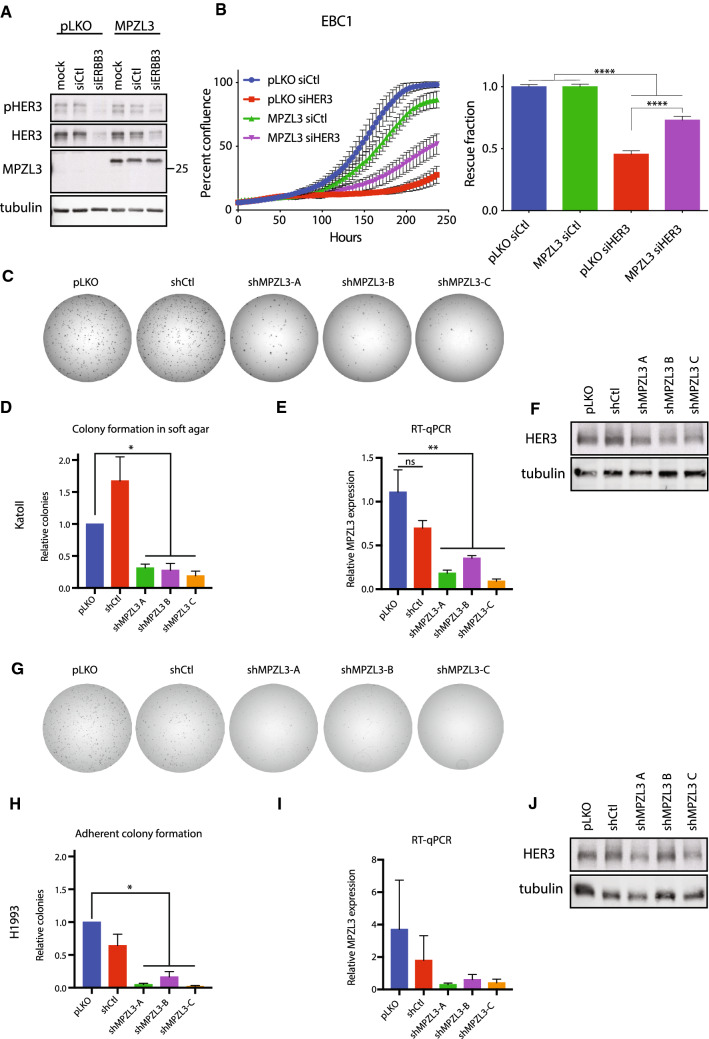
MPZL3 is required for *MET*-amplified cell expansion downstream of HER3. **a** Western blot analysis of total and phosphorylated levels of HER3 and MPZL3 in EBC1 cells overexpressing MPZL3, or an empty-vector control (pLKO) and transfected with control or HER3*-*targeting siRNAs. **b** Live-cell imaging to measure cell confluence of EBC1 cell lines stably transduced with control (pLKO) or MPZL3 and transfected with control or HER3*-*targeting siRNAs. Representative growth curves are shown; Error bars indicate SEM. Confluence relative to relevant siCtl-treated cells is quantified on the right panel (*n* = 3); error bars show SEM. **c**, **g** Representative images of colony-forming assay are presented. **c**, **h** Colony formation of KatoII cells in soft agar (**c**–**d**) or H1993 cells in adherent conditions (**g**–**h**) transduced with shRNA targeting MPZL3 or controls (pLKO and shCtl). Quantification of the colony number (*n* = 4). **e**, **i** MPZL3 transcript levels in KatoII (**e**) and H1993 (**i**) cells transduced with shRNA targeting MPZL3 or controls (pLKO and shCtl). **f**, **j** Western blot analysis of total and phosphorylated levels of HER3 in KatoII **f** and H1993 **j** cells transduced with shRNA targeting MPZL3 or controls (pLKO and shCtl). **b**: One-way ANOVA with the Sidak–Holm test for multiple comparisons; ****p ≤ 0.0001. **d**–**e** and **h**–**i** : One-way ANOVA with Dunnett’s test for multiple comparisons; *p ≤ 0.05; **p ≤ 0.01

We tested the ability of MPZL3 to rescue HER3-dependent proliferation by overexpressing MZPL3 in EBC1 cells with or without knockdown of HER3. Empty-vector control and MPZL3-overexpressing EBC1 cells were then transfected with pooled siRNA targeting ERBB3 or control duplexes (Fig. 4a). Cell proliferation in HER3-depleted cells was compared to control-siRNA-treated cells with or without overexpression of MPZL3. Cells overexpressing MPZL3 were significantly less sensitive to decrease in proliferation observed following HER3 (*ERBB3*) depletion when compared to control cells (0.45 ± 0.029 for pLKO-siHER3/pLKO-siCtl cells versus 0.73 ± 0.031 for MPZL3-siHER3/pLKO-siCtl) (Fig. 4b). Thus, MPZL3 overexpression partially overcomes the cell expansion defect following HER3 knockdown in MET-amplified EBC1 cells.

To further test the contribution of MPZL3 to *MET*-amplified cells, we depleted MPZL3 using shRNA in cell lines in our panel. KatoII cells were impaired in their ability to form colonies in soft agar, as observed for HER3 knockdown cells (Figs. 4c–f). We monitored H1993 *MET*-amplified cell lines depleted for MPZL3 with shRNA by clonogenic assay under adherent conditions and observed a dependence on MPZL3 similar to that observed following HER3 depletion (Figs. 4g–j). Together, these observations support a role for MPZL3 for proliferation in *MET*-amplified cells, and that HER3-dependent MPZL3 levels may underlie a requirement for HER3 for robust proliferation in *MET-*amplified cancers.

### HER3 and MPZL3 proteins interact

**Fig. 5:**
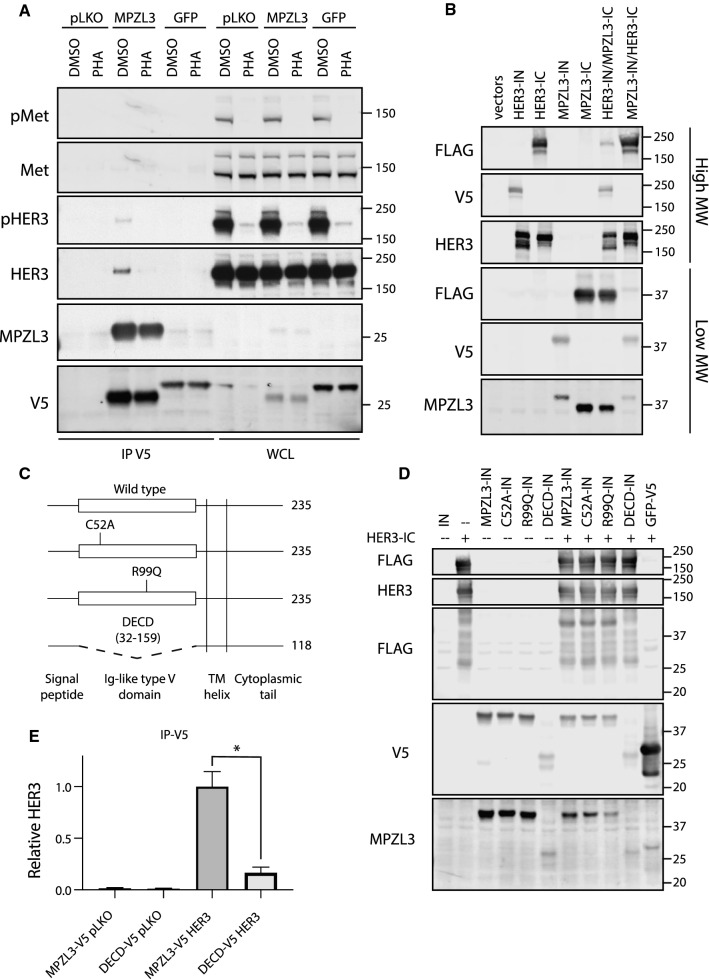
HER3 interacts with MPZL3. **a** Co-immuno-precipitation analysis of KatoII cells stably transduced with MPZL3- or GFP-V5 expression vectors, or control (pLKO). Cells were treated with PHA-665752 (0.5 mM, 1 h) or with vehicle control (DMSO). Immunoprecipitation (IP), Whole cell lysate (WCL). **b** HER3 and MPZL3 were analyzed by split intein-mediated protein ligation (SIMPL) assay to measure direct protein–protein interaction. **c** Schematic of MPZL3 mutant constructs. **d** FLAG transfer by point mutants and the ΔECD mutant were compared to wild-type MPZL3 using the SIMPL assay. **e** Co-immunoprecipitation of MPZL3 and HER3 was quantified, comparing HER3 recovered by the full-length or ΔECD MPZL3 mutants. Data are means (bars) of three individual replicates. Student’s *t* test; *p ≤ 0.05; **p ≤ 0.01; ***p ≤ 0.001; ****p ≤ 0.0001. Error bars indicate SEM

While our results demonstrate that HER3 depletion significantly reduces MPZL3 mRNA, we hypothesized that MPZL3 protein may interact with Met or HER3. To investigate this, we transduced KatoII *MET*-amplified cells with lentiviral vectors encoding MPZL3-V5, GFP-V5 and an empty-vector controls. Under these conditions MPZL3-V5 co-immuno-precipitated with HER3 but not with Met. Interestingly, this interaction was reduced by Met inhibition using PHA (37.9% of DMSO control ± 11.2%) (Fig. 5a). This supports that MPZL3 interacts with HER3 and further indicates that the HER3–MPZL3 interaction can be modulated by Met activity in *MET*-amplified cells.

To test whether MPZL3 could interact directly with HER3, we used the newly developed Split Intein-Mediated Protein Ligation (SIMPL) technique. SIMPL employs Intein N (IN) and Intein C-terminal (IC) fragments of a self-splicing intein domain fused to bait and prey proteins. When the two fused proteins interact, the intein protein activates a protein splicing reaction, resulting in the fusion of the two test proteins [45]. The co-expression of MPZL3-IC (FLAG- tagged) and HER3-IN (V5-tagged) in HEK293T cells resulted in a fusion of the FLAG tag to the V5-tagged bait protein as captured as a spliced band co-migrating with HER3 or MPZL3 recognizable by FLAG and V5 antibodies (Fig. 5b). This indicates that MPZL3 and HER3 can interact in the absence of an amplified Met RTK.

We investigated by structure-function analyses MPZL3 protein domains required for this interaction and tested whether we could uncouple the HER3–MPZL3 interaction by mutating the single, extracellular Ig-like domain in MPZL3. We introduced point mutations in the extracellular domain predicted to impair the binding function of the Ig-like domain or deleted the entire Ig-like domain from MPZL3 (Fig. 5c). Mutation of cysteine 52 to alanine (C52A) impairs the formation of a highly conserved disulfide bond characteristic of Ig-like domains and is required for homophilic binding of the MPZL3 paralogue myelin protein zero [46]. A spontaneous arginine-to-glutamine substitution in the extracellular domain of murine *MPZL3* (R99Q in human MPZL3) has been shown to induce progressive hair loss in mice, and this effect is phenocopied by homozygous deletion of *MPZL3*, indicating that this mutation impairs  the function of MPZL3 under physiological conditions [43, 44, 47]. Using the SIMPL assay, we observed comparable FLAG transfer when wild-type, C52A or R99Q MPZL3 constructs were used as bait, but observed a loss of this effect upon deletion of the extracellular domain of MPZL3 (MPZL3-ΔECD) (Fig. 5d). Deletion of the extracellular domain also strongly impaired co-immunoprecipitation of HER3 and MPZL3 upon their co-overexpression in HEK293T cells (Fig. 5e). This supports a HER3–MPZL3 interaction dependent on the extracellular domain of MPZL3 which can occur independent of Met amplification and Met-dependent HER3–tyrosine phosphorylation, yet can be potentiated by Met activity in Met-amplified Met-dependent cells.

### MPZL3 is overexpressed in MET-, EGFR- and ERBB2-amplified cancer cell lines

While we have observed a role for MPZL3 in a HER3-dependent proliferative response in MET-amplified cells, it is unclear whether this is a specific requirement of Met-dependent transformation independent of HER3 or whether MPZL3 expression is associated with RTK amplification in cancer. To understand if MPZL3 expression co-occurs in cancers with amplified RTKs, we interrogated the Cancer Cell Line Encyclopedia (CCLE) genomic copy number and gene expression data to explore the relationship between RTK amplification and MPZL3 expression (copy relative to ploidy + 1 ≥ 2.3) [48–50]. Of the 1280 cell lines with data available, 98 (7.8%) had at least one RTK gene amplified. Notably, none of the cell lines with amplification of an RTK also showed amplification of the MPZL3 gene at this threshold, indicating that any relationship of MPZL3 RNA with an amplified RTK may be primarily under transcriptional regulation.

**Fig. 6:**
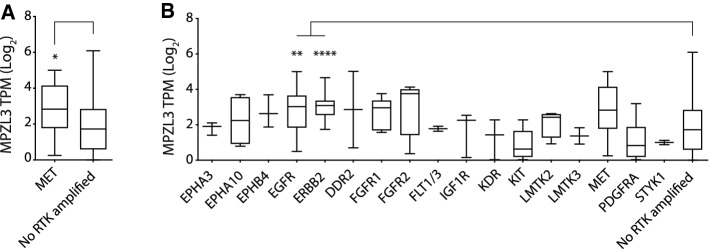
Amplification of genes encoding *MET*, *EGFR* and E*RBB2* correlate with elevated levels of *MPZL3* in the Cancer Cell Line Encyclopedia. **a** Met-amplified cells were identified in the CCLE by relative gene copy number (Log_2_ relative to ploidy + 1 ≥ 2.3), and MPZL3 expression was observed to be higher than that in cells with no RTK amplified (median TPM: 2.8 [*MET*-amplified] vs. 1.7 [control], Mann–Whitney *p* = 0.03). **b** Cell lines in CCLE were profiled for RTK amplification (Log_2_ relative to ploidy + 1 ≥ 2.3), and MPZL3 expression was compared in cells with an amplified RTK to cells with no RTK amplification. ANOVA *p* < 0.0001

Examination of 11 cell lines presenting MET amplification (copy relative to ploidy + 1 ≥ 2.3) revealed a significant increase of MPZL3 at the transcript level when compared to cell lines with no RTK amplified (1160 cell lines) (Fig. 6a). To determine whether transcriptional elevation of MPZL3 might be associated with RTKs other than Met, we analyzed all 27 cell lines with identified amplified RTKs. Comparing across all RTKs amplified in multiple CCLE cell lines available, EGFR and ERBB2 amplification significantly predicted elevated expression of MPZL3 relative to cells with no amplified RTK (Fig. 6b). Thus, the CCLE copy number and gene expression data support the elevation of MPZL3 mRNA levels downstream of multiple amplified RTKs in human cancer-derived cell lines.

### MPZL3 level correlates with MET and ERBB3 expression and is associated with poor clinical outcome in gastric cancer

**Fig. 7:**
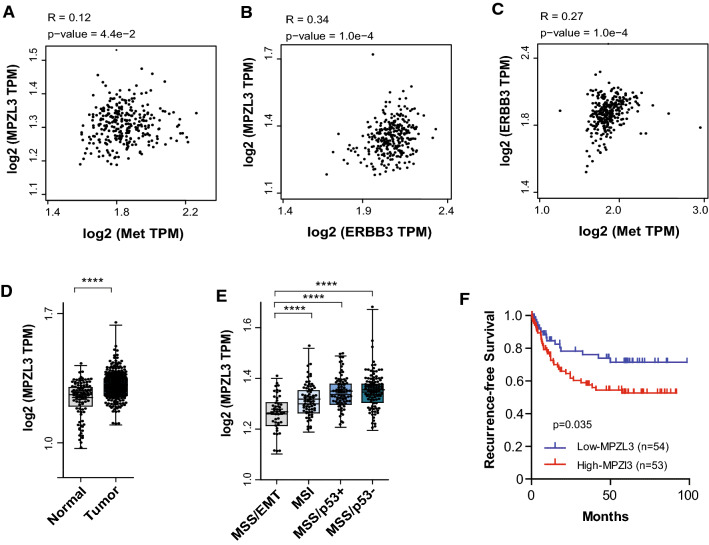
*MPZL3* levels correlate with *MET* and *ERBB3* gene expression and are associated with poor clinical outcome in gastric cancers. **a**–**c** Correlation between the indicated genes in gastric tumors, estimated using Pearson’s correlation (*n* = 300). **d** Expression of *MPZL3* in normal gastric tissues (*n* = 100) and gastric tumors (*n* = 300). **e** Expression of *MPZL3* across gastric cancer subtypes. EMT, Epithelial-mesenchymal transition (*n* = 46); MSI, microsatellite instability (*n* = 68); MSS, microsatellite stable; TP53^–^, TP53 loss (*n* = 107); TP53^+^, TP53 intact (*n* = 79). **f** Recurrence-free survival across gastric intestinal adenocarcinoma according to Lauren classification (*n* = 107). Data from GSE66229. Student’s *t* test; *p ≤ 0.05; **p ≤ 0.01; ***p ≤ 0.001; ****p ≤ 0.0001

To further investigate the relevance of Met and HER3 in MPZL3 regulation, we examined available datasets of gastric [51] and lung squamous cell carcinomas (LUSC) [52]. We found that *MPZL3* expression is positively correlated with *MET* and *ERBB3* expression in gastric tumors (Fig. 7a–c) and in LUSC (Supplementary Fig. 5a–b). We discovered that *MPZL3* mRNA is significantly increased in gastric cancer when compared to normal gastric tissues (Fig. 7d). Gastric adenocarcinoma is an aggressive and poorly understood cancer with a heterogeneous tumor biology and presentation. Recent advances in molecular biology have better defined gastric cancer subtypes using molecular subtype. Using the ACRG and TCGA classification [53–55], we found that *MPZL3* is elevated in tumors with the Microsatellite stable/ TP53 loss (MSS/p53^−^) subtype, a subgroup associated with a less-favorable prognosis compared to Microsatellite instability (MSI) and MSS/epithelial/TP53 intact subtypes [53, 55]. In agreement with this observation, elevated levels of *MPZL3* are associated with reduced recurrence-free survival in gastric and LUSC (Fig. 7f and Supplementary Fig. 5c).

## Discussion

Cancers harboring high-level amplification of the *MET* gene, with more than five copies of *MET* for each copy of chromosome seven, have been known to enrich for response to Met inhibitors in the clinic [56–59]. *MET* amplification and overexpression correlate with constitutive activation of the Met RTK and downstream signaling pathways in the absence of the Met ligand, HGF, in cancer-derived cell lines, indicating that the induction of constitutive Met signaling underlies this clinical observation [20, 21]. By analyzing preference within the EGFR family for crosstalk with Met in *MET*-amplified cell lines, we have uncovered a critical role for HER3 in Met-dependent oncogenic signaling and have shown that this involves the expression and recruitment of a novel HER3-dependent transcript and interactor, MPZL3.

We found that while EGFR, HER2 and HER3 were co-expressed and usually tyrosine phosphorylated in our panel of *MET*-amplified cell lines, HER3 phosphorylation consistently showed dependence on Met activity, while EGFR and HER2 were frequently phosphorylated independently of Met. Supporting our biochemical observations that a Met–HER3 axis recurrently arises in *MET*-amplified cells, we found that HER3 expression was important for core oncogenic processes downstream of Met in the three independent *MET*-amplified tested cell lines. Collectively, these results demonstrate a convergent selection for crosstalk between HER3 and the Met signaling pathway in Met-dependent cancers. Furthermore, our analysis of upstream kinases shows that while HER2 kinase activity is required for HER2 phosphorylation in most *MET*-amplified cell lines, this has no impact on HER3 phosphorylation, indicating co-option of HER3 signaling by Met in the presence of an intact HER2 signaling module, together suggesting that a HER3-dependent signaling axis plays an important role in *MET*-amplified cells and is under strong selection for Met-dependent activation in this context.

HER3 has been shown to play a critical role in EGFR- and HER2-dependent cancers, including cancer cells harboring an EGFR mutation or amplification of the *ERBB2* gene [15, 26, 60]. Intriguingly, in EGFR-mutant HCC827 lung cancer cells subjected to prolonged gefitinib treatment in tissue culture, an acquired resistance mechanism involved *MET* gene amplification, Met-dependent HER3–tyrosine phosphorylation, and HER3-dependent activation of PI3K downstream of Met [26]. *MET* amplification has been observed in lung cancer patients treated with the EGFR inhibitors gefitinib and erlotinib, and treatment of gefitinib-resistant tumors with the Met inhibitor crizotinib is effective in the clinic for these patients [28]. While this has been suggested to depend on activation of the PI3K signaling pathways including the Akt pathway, in our panel of *MET*-amplified cells, PI3K activity-associated phosphorylation of Akt remained intact following inhibition of Met kinase with small molecule inhibitors. We similarly did not observe a significant increase in apoptosis in EBC1, H1993 and KatoII *MET*-amplified cells upon depletion of HER3, or changes in known Akt-dependent gene expression (data not shown), supporting the idea that Met-dependent activation of pro-survival signaling downstream of the Akt pathway remained intact. This, in turn, indicated that the contribution of HER3 to Met-dependent signaling proceeded through a novel HER3-dependent mechanism, and did not impact the survival pathways canonically associated with oncogenic HER3 signaling.

The highest confidence and most differentially expressed candidate genes downstream of HER3 were genes whose expression decreased upon HER3 knockdown, including *MPZL3*, a gene encoding a transmembrane protein with an extracellular Ig-like domain and an unstructured cytoplasmic tail involved in epidermal differentiation [44, 61]. Our observations that suppression of MPZL3 phenocopies HER3 knockdown and that MPZL3 overexpression can partially rescue proliferation upon HER3 knockdown support a role for MPZL3 downstream of HER3 in *MET*-amplified cell lines. This would explain the recurrence of Met–HER3 crosstalk in *MET*-amplified cells, as a proliferative advantage would lead to convergent selection for this signaling axis.

MPZL3 was initially identified as a regulator of skin morphogenesis via a spontaneous point mutation, leading to arginine-to-glutamine (R99Q in human MPZL3) amino acid substitution in the extracellular Ig-like domain [43]. Knockout *Mpzl3* mice models showed impaired differentiation of sebaceous gland precursor, leading to alteration of skin development, hair growth and adipocytes functions [43, 44, 47]. More recently, MPZL3 has been identified as a radio-resistant candidate marker gene in rectal cancer cell lines [62]. However, the role of MPZL3 in cancer progression and its regulation remains poorly defined. Our co-immunoprecipitation and SIMPL assay experiments demonstrate that the extracellular Ig-like domain is required for the formation of a HER3–MPZL3 complex. The C52A and R99Q mutations, however, do not impair the association of MPZL3 with HER3, implying that different functions of the Ig-like domain are required for MPZL3-dependent epidermal differentiation and its interaction with HER3.

While our experiments in HEK293T cells demonstrate an association between MPZL3 and HER3 in the absence of Met amplification or activation, we observe a loss of this interaction when Met kinase activity is inhibited by PHA in MET gene-amplified and MET-dependent KatoII cells. This may be due to a direct effect of Met activity on the protein interaction between HER3 and MPZL3 or proceed via an indirect mechanism, such as altered trafficking of either HER3 or MPZL3 in a Met-dependent manner in MET-amplified cells.

Taken together, our results support that MET amplification co-opts HER3 and its binding partner MPZL3 to support oncogenic cell proliferation via a mechanism previously undescribed. Accordingly, MET gene amplification predicted higher *MPZL3* expression than cell lines without any RTK amplification and *MPZL3* levels are positively correlated with MET and ERBB3 expression in LUSC and gastric carcinomas. While MPZL3 has been reported to act as a tumor suppressor in cutaneous squamous cell carcinoma through its activity promoting differentiation [63], our proliferation and colony-forming assays demonstrate an oncogenic role for *MPZL3* in lung and gastric carcinoma cells. In agreement with this, *MPZL3* is strongly enriched in gastric adenocarcinomas, particularly in the MSS/p53^−^ subtype, and is associated with poorer clinical outcome. These observations are of importance as they are to our knowledge, the first to study the clinical relevance of MZPL3 in gastric and lung cancers, further supporting the therapeutic potential of the HER3–MPZL3 axis in *MET*-amplified cancers.

Convergence on a shared HER3–MPZL3 dependency could explain the observation that Met and the EGFR family receptors are important bypass pathways for each other in a number of models of resistance to EGFR or HER2 inhibition, as reactivation of any of these receptors could stabilize MPZL3 levels in a similar manner [26–29, 64]. As MPZL3 and HER3 interact directly, it may be possible to identify cancers dependent on this interaction and the upstream activity of HER3-activating kinases, such as Met, EGFR and HER2, via this mechanism. Intriguingly, HER3 phosphorylation has been repeatedly observed as a bypass mechanism of resistance to BRAF and MEK1/2 inhibitors in melanoma, indicating that there may be additional mechanisms in which HER3 plays a critical and central role in acquired resistance [65–68]. Further analysis of the emerging biology of HER3 and MPZL3 and their activities in human cancer will be critical to fully exploiting their potential in cancer therapy.

## Materials and methods

### Antibodies and reagents

Antibody 148 was raised in rabbit against a C-terminal peptide of human Met [69]. Antibodies against phosphorylated Met at Tyr^1234/1235^, HER2 at Tyr^1221/1222^, HER3 at Tyr^1289^, HER3 at Tyr^1222^, Akt at Ser^473^, Erk1/2 at Thr^202^/Tyr^204^, STAT3 at Tyr^705^, and generic tyrosine peptide (pY100), as well as total EGFR, HER2, HER3, pan-Akt, Erk1/2, and STAT3 were purchased from Cell Signaling Technologies. Antibodies against phosphorylated EGFR at Tyr^1173^ were purchased from Santa Cruz Biotechnology. Antibodies against M2-FLAG peptide, actin and tubulin were purchased from Sigma. Antibodies against V5 peptide were purchased from Abcam, while antibodies against MPZL3 were purchased from ProteinTech. HRP-conjugated secondary antibody was purchased from Cell Signaling, while IRDye infrared secondary antibodies were purchased from Mandel Scientific. Details of antibodies are provided in Supplementary table 1.

PHA-665752 (Met inhibitor) was used at 0.5 µM and was a gift from Pfizer. Gefitinib and lapatinib, used at 1 µM, and dasatinib, used at 100 nM, were gifted by Dr. W. Muller. Tepotinib used at 0.1 µM, was from Cedarlane. Crizotinib used at 0.1 µM, was from

Active Biochemicals. Dimethyl sulfoxide (DMSO) was purchased from Sigma and used at a concentration of 1:1000 as a vehicle control.

### Cell cultures and RNAi

OE33 cells were described previously [31] and cultured in RPMI supplemented with 10% FBS and 2 mM L-glutamine (Thermo Fisher). Snu5, KatoII, Okajima, and MKN45 cells were cultured as described previously [21, 70, 71]. EBC1 were obtained through RIKEN BRC Cell Bank and H1993 cells were obtained from ATCC; these were cultured in MEM alpha or RPMI media, respectively, supplemented with 10% FBS (Thermo Fisher). SkBr3 cells were gifted by Dr. W. Muller and were cultured in McCoy’s 5A media supplemented with 10% FBS (Themo Fisher). HEK293T cells were cultured in DMEM supplemented with 10% FBS (Thermo Fisher).

EBC1 cells were transfected in suspension using pooled siRNA at 50 nM using the HiPerfect protocol (Qiagen) and immediately seeded for proliferation and lysis, which was conducted after 72 h. Allstars control siRNA was purchased from Qiagen. siGENOME SMARTpool pooled siRNA duplexes targeting *ERBB3* were purchased from Dharmacon (sequences in Supplementary table 2). Viral vectors for shRNA expression as well as empty-cassette plasmids used as vector controls were obtained from the Mission TRC library (Sigma) via the McGill Platform for Cellular Perturbation (clone information and sequences in Supplementary table 3). Gateway entry vector for MPZL3 (clone information and sequence in Supplementary table 4), Gateway destination vectors pLX303, pLX304, and pLEX307 for mammalian cDNA overexpression, and pLX317 vector for GFP-V5 overexpression were obtained through the Mission TRC3 Orfeome collection via the McGill Cellular Perturbation Services. Split intein-mediated protein ligation (SIMPL) Gateway destination vectors were from Stagljar lab [45]. Gateway entry vector for ERBB3 was obtained through Addgene (pDONR223-ERBB3, cat. 23,874). Vector recombination was performed using the Gateway LR Clonase II enzyme kit (Thermo Fisher).

### Lentiviral transduction

Transfections of HEK293T cells for viral production were performed by the calcium phosphate method. Lentiviral cultures for shRNA or transgene overexpression were collected as described at https://portals.broadinstitute.org/gpp/public/resources/protocols. Lentiviral transduction was followed by selection in puromycin (1–2 mg/ml) or blasticidin (2–10 mg/ml) for 3 and 14 days, respectively, and control cells were verified for negative selection prior to the start of each experiment.

### Proliferation and soft agar experiments

Proliferation assays were performed in 96-well dishes with 4× 10^3^ EBC1, H1993 or KatoII cells seeded per well with 18 replicates per condition. Proliferation was measured as a function of cell confluence by IncuCyte live-cell microscopy (Essen Biosciences). Soft agar assays were performed in 6-well dishes coated with a layer of bottom agar (6 mg/ml), with 1 × 10^4^ KatoII cells seeded in top agar (3.2 mg/ml) as previously described [72]. Cells were plated in duplicate per condition and fed every 4–5 days, with colonies counted after 23 days in culture. For both proliferation and soft agar assays, representative examples are shown of three independent replicates. Quantification was performed from all three replicates where shown.

### Subcutaneous xenograft experiments

For xenograft experiments, 5 × 10^5^ (KatoII) or 1 × 10^6^ (H1993) cells were injected bilaterally into the flanks of immune-deficient NOD.Cg-Prkdc^scid^Il2rg^tm1Wjl/SzJ^ mice (Jackson). Five mice were injected per condition. Tumors were detected by palpation and measured every 3 days. Animals injected with control KatoII cells were sacrificed for ethical considerations at 13 days post injection and mice injected with the KatoII Her3-depleted sacrificed at 18 days post injection.

### Flow cytometry

EBC1, H1993 or KatoII cell lines transduced with shRNA targeting HER3 or vector control were seeded in equal numbers and grown for 3 days before washing in PBS, followed by analysis of cell surface Annexin-V and cell permeability using the Annexin-V-FLUOS staining kit (Sigma). This experiment was conducted under conditions identical to the proliferation assays shown in Fig. 2. Flow cytometry was performed on live cells using a BD FACS Calibur cytometer at the McGill Life Sciences Complex Flow Cytometry Facility.

### Immunoprecipitation and Western blotting

Cells were harvested for protein lysis in a Triton–glycerol–HEPES (TGH) buffer described previously [70]. Lysates were frozen, thawed and centrifuged at full speed in an Eppendorf micro-centrifuge for 15 min and protein content was assessed by Bradford assay (Biorad). Cell lysate equivalent to 1 mg protein collected 36 h post-transfection was diluted to 500 ml in TGH buffer for each immunoprecipitation reaction and precleared with 10 ml sepharose protein G beads (GE Healthcare). Immunoprecipitation was performed overnight by incubation with anti-V5 antibody, followed by capture using sepharose protein G beads. Beads were washed three times in lysis buffer prior to analysis. Centrifuged whole cell lysate or immuno-precipitated proteins captured on sepharose beads were boiled for 5 min in Laemmli buffer. Samples were resolved using 8% or 12% SDS-PAGE (BioRad) or 4–12% gradient NuPAGE Protein Gel (Thermo Fisher). Proteins were transferred to Newblot PVDF membranes (Mandel Scientific), blocked in 3% bovine serum albumin in TBST or Odyssey Blocking Buffer (Licor) and incubated with primary antibodies diluted in Near-Infra-Red Blocking Buffer (Rockland) overnight at 4° C. Membranes were washed in TBS Tween (TBS-T) and incubated with secondary antibodies diluted in a 1:1 TBS-T:Rockland blocking buffer for one hour, washed in TBS-T again, and scanned using Odyssey scanning machine (Licor) or visualized using Western Lightning Plus ECL (Perkin Elmer). Membranes were stripped prior to reblotting with other antibodies using the stripping buffer, Thermo Scientific Restore Plus Western Blot Stripping Buffer (Thermo Fisher cat. PI46430) according to manufactures’ instruction.

### RNA sequencing

KatoII cells transduced with shHER3A, shHER3B or the pLKO empty vector were selected in puromycin and plated for experiments. Total RNA was extracted from 3 independent infections used to prepare replicates for soft agar and xenograft injection assay using the RNeasy Plus Mini Kit (Qiagen). High RNA quality in all samples was verified using the Bioanalyzer RNA Nano 600 assay (Agilent). cDNA libraries were PCR-amplified and barcoded from 500 nanograms of RNA per sample, and pooled libraries were sequenced using the HiSeq4000 next-generation sequencing platform (Illumina) by the Centre d’Expertise et de Services at Génome Québec. The pooled libraries were sequenced with an average coverage of 3.3 × 10^7^ reads per sample.

Fastp (version 0.19.4) was used to collect QC metrics of the raw reads. RNA sequences were aligned and sorted by coordinates, to the NCBI human genome build 38 (GRCh38.p12) with version 94 of gene annotations, using the STAR aligner (STAR_2.6.1a_08-27) [73, 74]. The removal of alignment duplicates was done with Sambamba (version 0.6.8) [75]. Quantification of genes was performed using featureCounts (v1.6.3) [76]. Differentially expressed genes among groups were identified using the R packages DESeq2 (v 1.20.0) [77] and Lima. After unpaired analysis, only genes with False Discovery Rate (FDR) < 0.05, and log2 fold change ≥ 1.0 were considered. The HGNC symbols were extracted and added to the DESeq2 results data frame using biomaRt (version 2.36.1) using the “hsapiens_gene_ensembl” dataset and the “Ensembl Release 94 (October 2018)” mart [78, 79]. Illumina sequencing data for RNA are available at GEO as GSE189461.

### Bioinformatic analysis

Bioinformatic analyses were performed using the expression data sets GSE189461, GSE66229 [51], Gepia2 [52] and the CCLE genomic copy number and gene expression data sets [48–50]. Statistical significance was assessed using a two-tailed Student’s *t* test, and ordinary one-way ANOVA with Tukey’s correction for multiple comparisons, unless otherwise indicated, using Prism software and correlative analysis by Pearson correlation.

### Quantitative RT-PCR

RNA was isolated from three independently infected and selected lines of EBC1 and H1993 cells using the RNeasy Plus Mini Kit (Qiagen). High RNA quality in all samples was verified using the Bioanalyzer RNA Nano 600 assay (Agilent). cDNA libraries were synthesized using the Transcriptor First Strand cDNA Synthesis Kit (Roche). qPCR reactions were performed using SYBR Green I Master on a LightCycler480 (Roche). Gene expression was normalized to the *GAPDH, HPRT* and *RPLP0* genes using the delta-delta Ct method. Primers for qPCR were purchased from Integrated DNA Technologies (primer information listed in Supplementary table 5).

### Statistical analysis

Quantitative data are presented as the means ± SEM. Statistical significance was assessed using a two-tailed Student’s *t* test, and ordinary one-way ANOVA with Tukey’s correction for multiple comparisons, unless otherwise indicated, using Prism software. Significance is as follows: *p* > 0.05, not significant (ns); ^∗^*p* ≤ 0.05; ^∗∗^*p *≤ 0.01; ^∗∗∗^*p* ≤ 0.001; ^∗∗∗∗^*p* ≤ 0.0001. Data distribution was assumed to be normal, but this was not formally tested. P values and the number of experiments used for quantification and statistical analysis are indicated in the corresponding figure legends.

## Supplementary Information

Below is the link to the electronic supplementary material.

Supplementary file1 (PDF 107066 kb)

## Declarations

### Availability of data and materials

The RNA sequencing dataset generated during the current study is available on the NCBI GEO platform (GSE189461). Other data are available at the relevant referenced web locations or are available from the corresponding author on reasonable request.
