# Supplementary material for: Met–HER3 crosstalk supports proliferation via MPZL3 in MET-amplified cancer cells
Source: Cell Mol Life Sci. 2022 Mar 5;79(3):178. doi: 10.1007/s00018-022-04149-w (PMC8898245; doi:10.1007/s00018-022-04149-w)
Supplement: Supplementary file 1 — Supplementary file1 (PDF 107066 kb) [file 18_2022_4149_MOESM1_ESM.pdf]

Met-HER3 crosstalk supports proliferation via MPZL3 in *MET*-amplified cancer cells

Yaakov E. Stern, Abdulhameed Al-Ghabkari, Anie Monast, Benoit Fiset, Farzaneh Aboualizadeh, Zhong Yao, Igor Stagljar, Logan A. Walsh, Stephanie Duhamel, Morag Park

Supplementary material

Supplementary Table 1: Antibodies

Antibody	Supplier	Catalogue number	Dilution
pMET Y1234/5 (D26) XP	Cell Signaling	3077	1:1000
MET	In-house	148	1:1000
pAKT S473 (D7F10) XP	Cell Signaling	9018	1:2000
AKT (40D4)	Cell Signaling	2920	1:1000
pERK1/2 T202/Y204	Cell Signaling	9101	1:2000
ERK1/2 (3A7)	Cell Signaling	9107	1:1000
pHER2 (Y1221/1222)	Cell Signaling	2243	1:1000
HER2	Cell Signaling	2165	1:1000
pHER3(Y1289)	Cell Signaling	4791	1:500
pHER3 (Y1222)	Cell Signaling	4784	1:1000
HER3 (D22C5) XP	Cell Signaling	12708	1:1000
pSTAT3(Y705)	Cell Signaling	9145	1:1000
STAT3	Cell Signaling	8768	1:1000
pEGFR(Y1173)	Santa Cruz	4407	1:1000
EGFR	Cell Signaling	4267	1:1000
pY100	Cell Signaling	9411	1:1000
MPZL3	ProteinTech	25513-1-AP	1:1000
Beta-Actin (AC-15)	Sigma	A5441	1:4000
M2-flag	Sigma	F3165	1:2000
V5	Abcam	ab27671	1:1000
Alpha-tubulin (B512)	Sigma	T5168	1:2000
anti-rabbit-HRP	Cell Signaling	7074	1:10000
anti-mouse IRDye 680 nm	Mandel Scientific	LIC-926-68070	1:10000
anti-rabbit IRDye 800 nm	Mandel Scientific	LIC-926-32211	1:10000

Supplementary Table 2: siRNA duplex sequences

Pooled siRNA duplex sequences (sense)
GCAGUGGGAUUCGAGAAGUG
AGAUUGUGCUCACGGGACA
GUGGAUUCGAGAAGUGACA
GCGAUGCUGAGAACCAAUA

Supplementary table 3: shRNA clone sequences

shRNA	Clone	Expressed sequence
pLKO (vector)	SHC001	
shCtl	SHC002	CCGGCAACAAGATGAAGAGCACCAACTCGAGTTGGTGCTCTTCATCTTGTTGTTTTTG
shHER3 A	TRCN0000194972	CCGGCTTCGTCATGTTGAACTATAACTCGAGTTATAGTTCAACATGACGAAGTTTTTG
shHER3 B	TRCN0000199364	CCGGCCTGTGCATGTGCTCTTATTGCTCGAGCAATAAGAGCACATGCACAGGTTTTTG
shMPZL3 A	TRCN0000137252	CCGGGCAGCCACACAGTATCAATATCTCGAGATATTGATACTGTGTGGCTGCTTTTTG
shMPZL3 B	TRCN0000167937	CCGGCCAGGGTGTTTATATCGTCTTCTCGAGAAGACGATATAAACACCCTGGTTTTTG
shMPZL3 C	TRCN0000167873	CCGGGAGATCATCAGTAAAGACTTTCTCGAGAAAGTCTTTACTGATGATCTCTTTTTG

Supplementary table 4: MPZL3 clone sequence

shRNA	Clone	Expressed sequence
pLKO (vector)	SHC001	
shCtl	SHC002	CCGGCAACAAGATGAAGAGCACCAACTCGAGTTGGTGCTCTTCATCTTGTTGTTTTTG
shHER3 A	TRCN0000194972	CCGGCTTCGTCATGTTGAACTATAACTCGAGTTATAGTTCAACATGACGAAGTTTTTG
shHER3 B	TRCN0000199364	CCGGCCTGTGCATGTGCTCTTATTGCTCGAGCAATAAGAGCACATGCACAGGTTTTTG
shMPZL3 A	TRCN0000137252	CCGGGCAGCCACACAGTATCAATATCTCGAGATATTGATACTGTGTGGCTGCTTTTTG
shMPZL3 B	TRCN0000167937	CCGGCCAGGGTGTTTATATCGTCTTCTCGAGAAGACGATATAAACACCCTGGTTTTTG
shMPZL3 C	TRCN0000167873	CCGGGAGATCATCAGTAAAGACTTTCTCGAGAAAGTCTTTACTGATGATCTCTTTTTG

Supplementary table 5: Sequences for RT-qPCR primers

Gene		Primers
ERBB3	forward	GGGGAGTCTTGCCAGGAG
	reverse	CATTGGGTGTAGAGAGACTGGAC
MPZL3	forward	ATTCCCATGACAGAGCTAACAG
	reverse	GCACAAAGACAAGGATGGAAAG
NYNRIN	forward	CTGAGCTGAGACCAGGATTAAG
	reverse	GTTGGAGAGAATGTGGGTGTAG
LGR6	forward	CTCTTCCCTTTCCTCTC
	reverse	CTGAGTTTTGGTTGTATTTG
BHLHE41	forward	AGAGGAAACGAACAGCAGTTGA
	reverse	TAGGTATCCTTGGTGTCGTCTCG
RAMP1	forward	TGGAGCCTTGGGACAGA
	reverse	GGCTTCCAGGTTAATACCAGAG
SPNS2	forward	CCATCTTCATCTGCCTGATCTT
	reverse	CAGTGATGGCCCAGTTAGAAA
PPM1K	forward	TTTCCAAACCAACAGGCAAAG
	reverse	CCTCCCAAAGTGCTAGGATTAC
RPLP0	forward	CTCAACATCTCCCCCTTCTC
	reverse	GACTCGTTTGTACCCGTTGA
GAPDH	forward	GCACCAGGTGGTCTCCTCT
	reverse	TGACAAAGTGGTCGTTGAGG
HPRT	forward	TGATAGATCCATTCCTATGACTGTAGA
	reverse	CAAGACATTCTTTCCAGTTAAAGTTG

Supplementary Figure 1

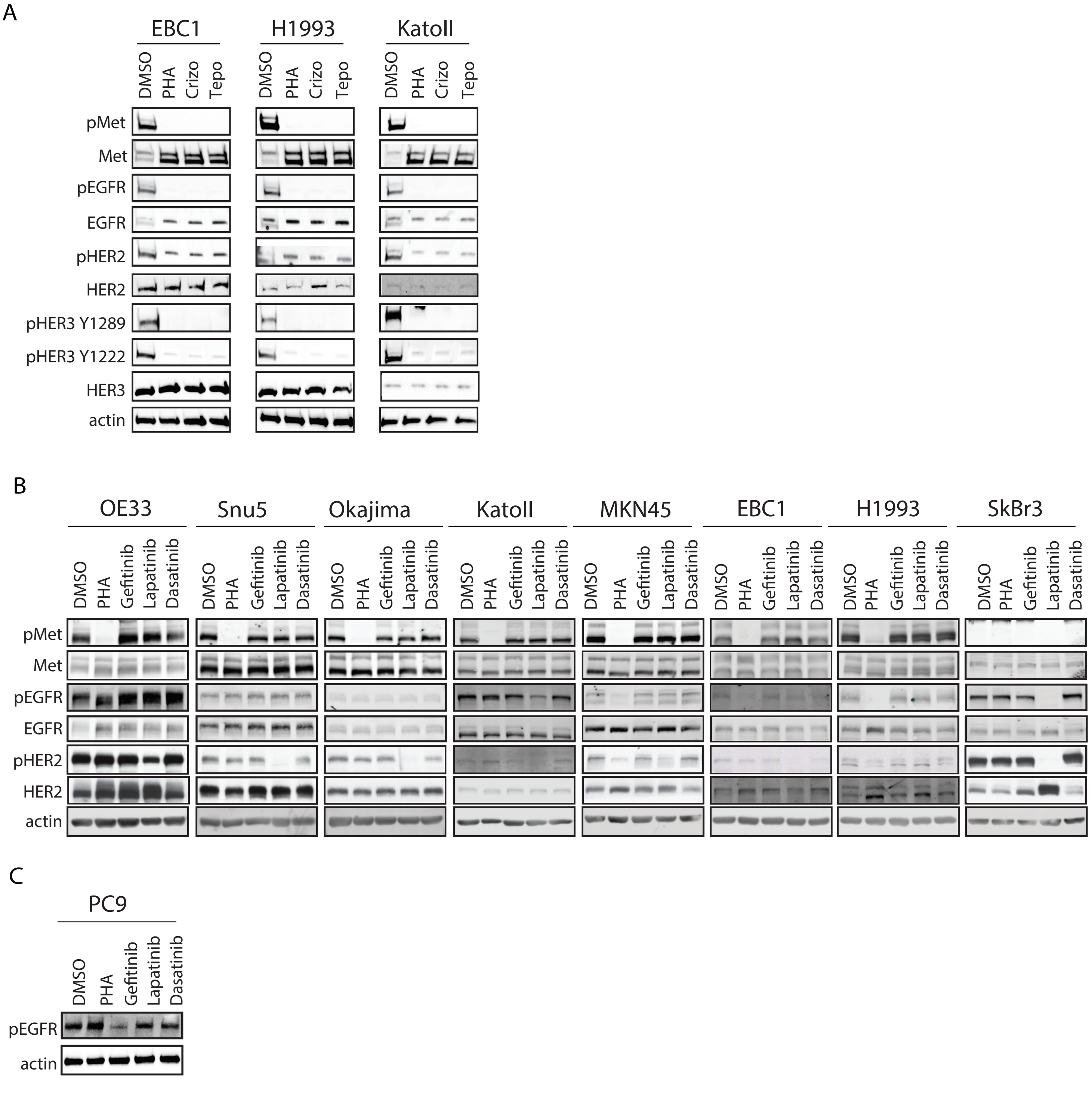

Supplementary Fig. 1 Controls for multiple kinase inhibitors used in MET-amplified cells. Controls for multiple kinase inhibitors used in MET-amplified cells. (A) Western blot analysis of the phosphorylation of Met (Tyr1234/35), EGFR (Tyr1173), HER2 (Tyr1221/22), HER3 (Tyr1289), and HER3 (Tyr1222) in the indicated cell lines treated with the Met inhibitors: PHA (0.5 μM); Crizotinib (Crizo) (0.1 μM); and Tepotinib (Tepo) (0.1 μM), for 1 hour (n=2). (B) Western blot analysis of the phosphorylation of Met (Tyr1234/35), EGFR (Tyr1173) and HER2 (Tyr1221/22) in the indicated cell lines treated with PHA (0.5 μM); the EGFR inhibitor, gefitinib (1 μM); the HER2/EGFR inhibitor lapatinib (1 μM); or the Src and Abl family kinase inhibitor, dasatinib (0.1 μM), for 1 hour (n=3). (C) Western blot analysis of the phosphorylation of EGFR (Tyr1173) in PC9 cells treated with PHA (0.5 μM); the EGFR inhibitor, gefitinib (1 μM); the HER2/EGFR inhibitor lapatinib (1 μM); or the Src and Abl family kinase inhibitor, dasatinib (0.1 μM), for 1 hour (n=2).

Supplementary Figure 2

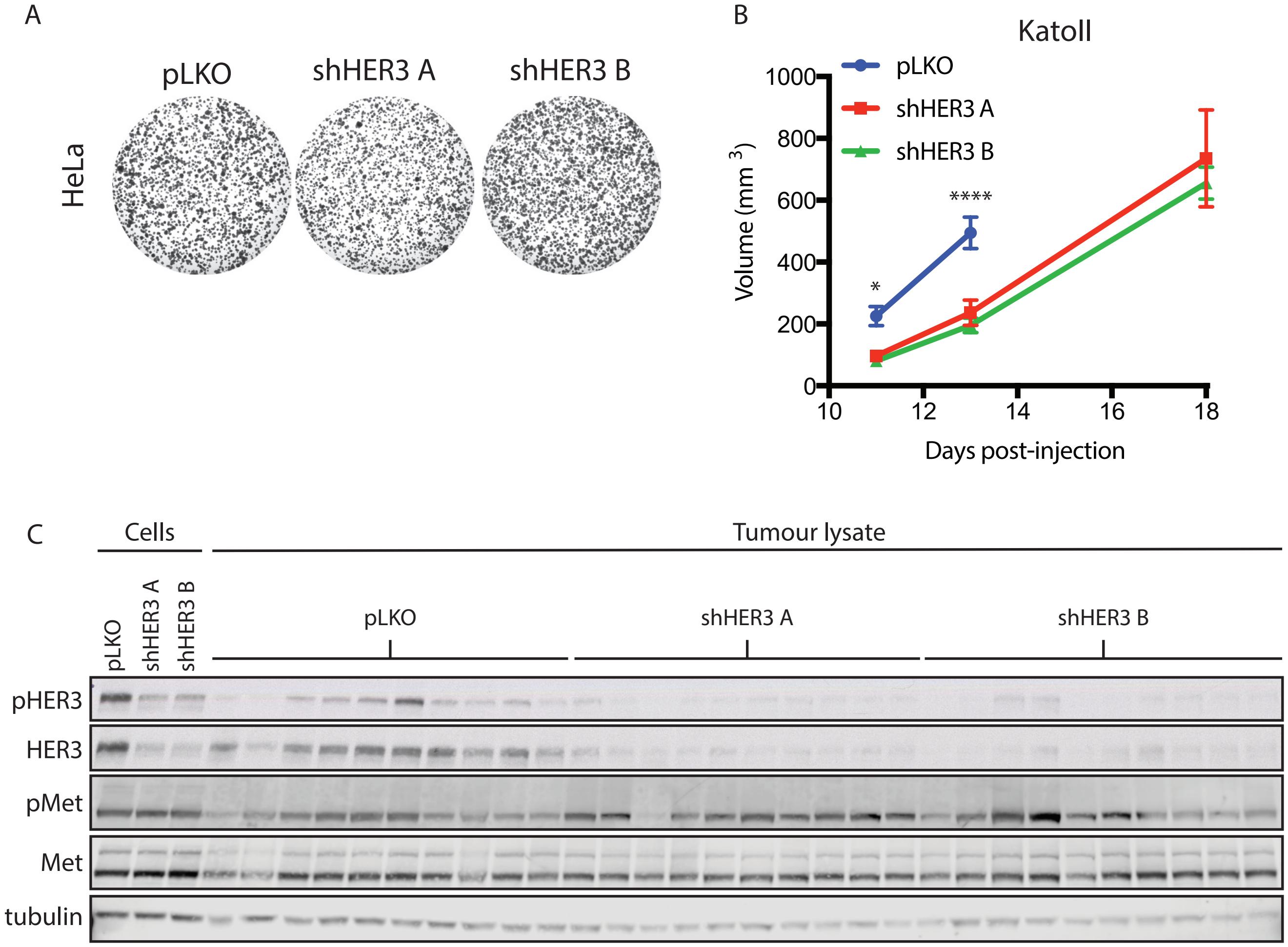

Suppplementary fig. 2 Growth of shRNA-treated cells in vitro and in vivo. (a) HeLa cells transduced with shRNA targeting HER3 form colonies at the same rate as controls (pLKO (n=3). (b) Tumour volumes of KatoII-derived xenografts shown in figure 2e at both points measured before collection (n=10). (c) Western blot analysis of total and phosphorylated Met and HER3 in KatoII tumours at end-point. *, $P < 0.05$, ****, $P < 0.0001$.

Supplementary Figure 3

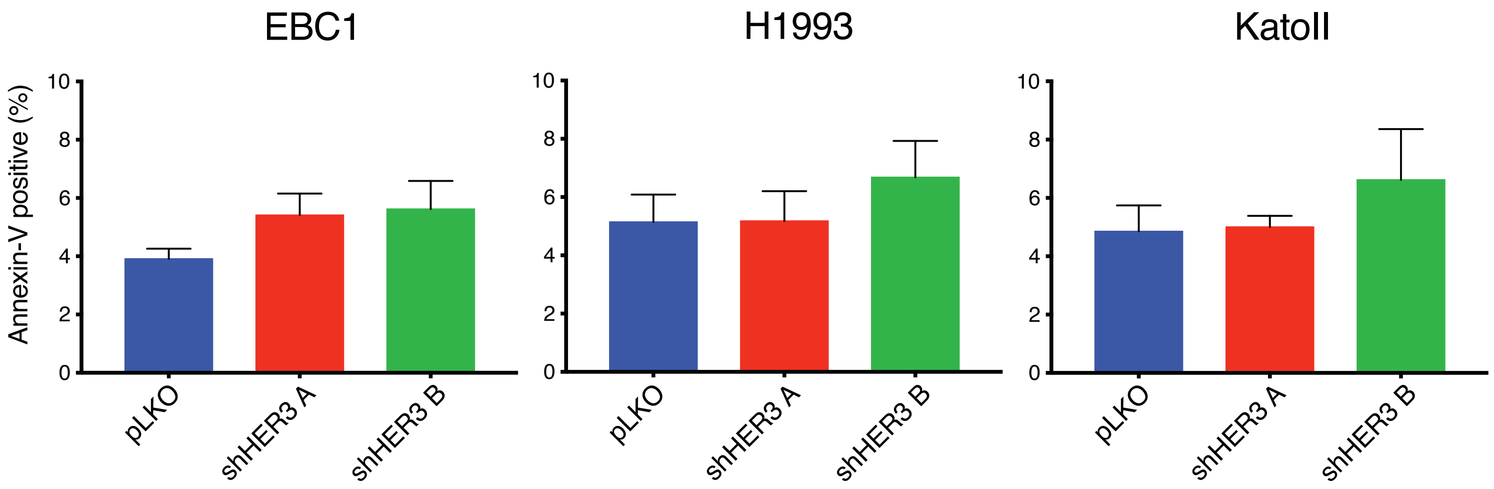

Supplementary Fig. 3 Apoptosis in shRNA-treated cells. Analysis of annexin-V positivity by flow cytometry in EBC1, H1993 and KatoII cells transduced with shRNA targeting HER3 or control (pLKO).

Supplementary Figure 4

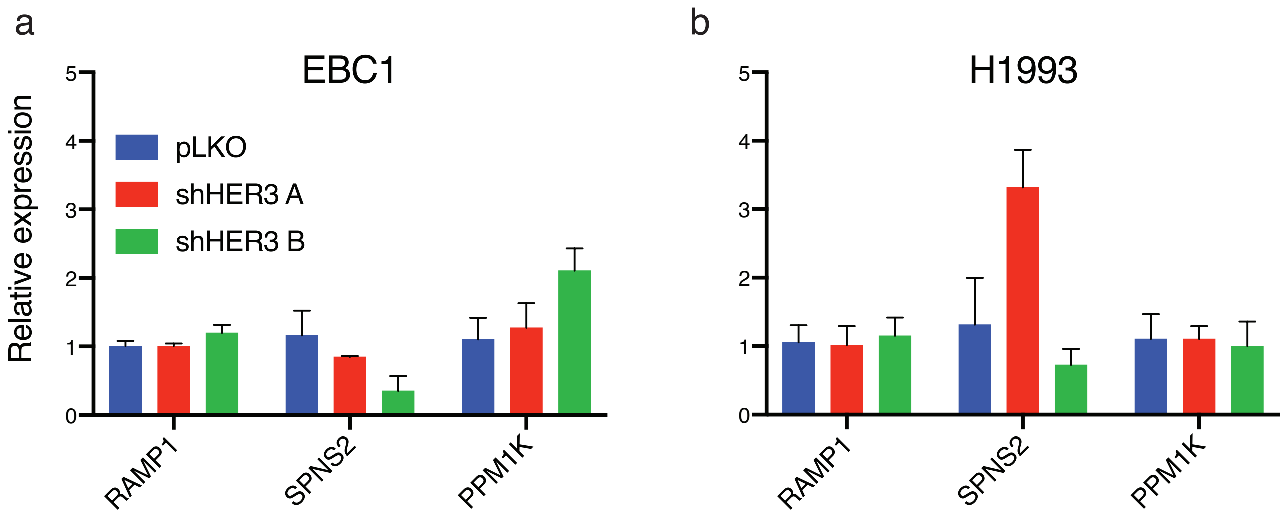

Supplementary Fig. 4 Gene expression analysis of upregulated transcripts from KatoII RNA sequencing in EBC1 and H1993 cells. Measurement of the relative amounts of RAMP1, SPNS2 and PPM1K genes in the indicated cell lines by RT-qPCR

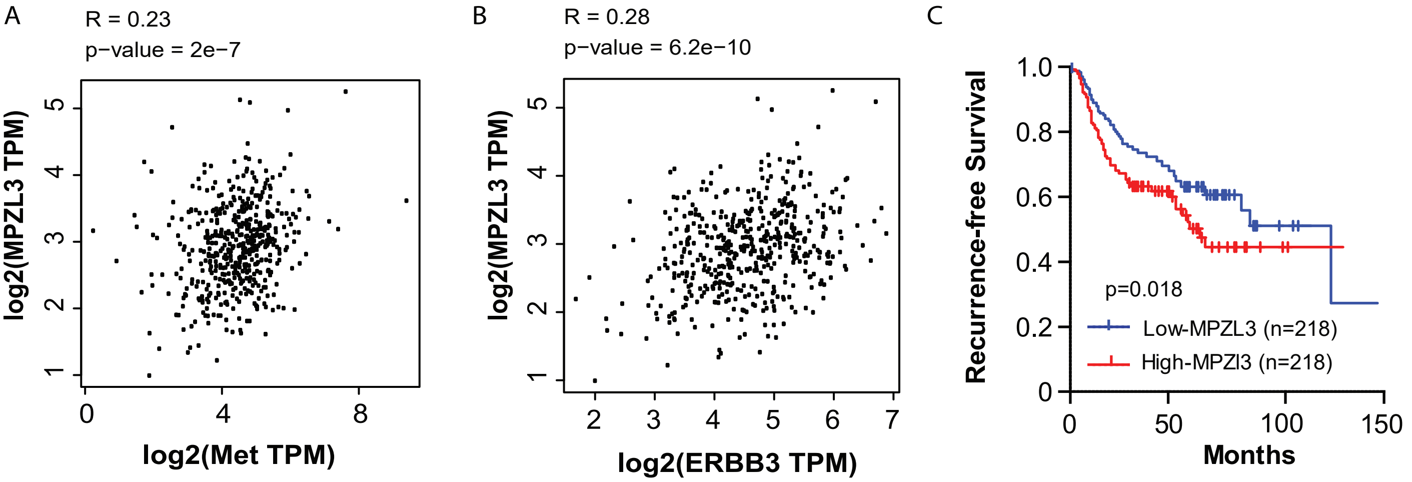

Fig. S5 MPZL3 levels correlate with Met and ERBB3 expression and are associated with poor clinical outcome in Lung squamous cell cacinomas. (a-b) Correlation between the indicated genes in LUSC tumors, estimated using Pearson's correlation (n=436). (c) Recurrence-free survival in LUCS (n=436). Data from Gepia2. Student's t-test; $*p \leq 0.05$; $**p \leq 0.01$; $***p \leq 0.001$; $****p \leq 0.0001$.

Figure 1A

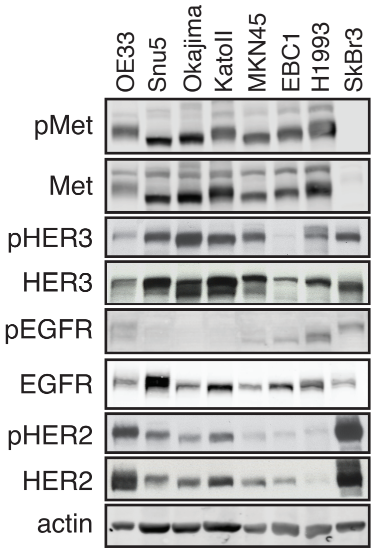
OE33
Snu5
Okajima
KatoII
MKN45
EBC1
H1993
SkBr3
pMet
Met
pHER3
HER3
pEGFR
EGFR
pHER2
HER2
actin

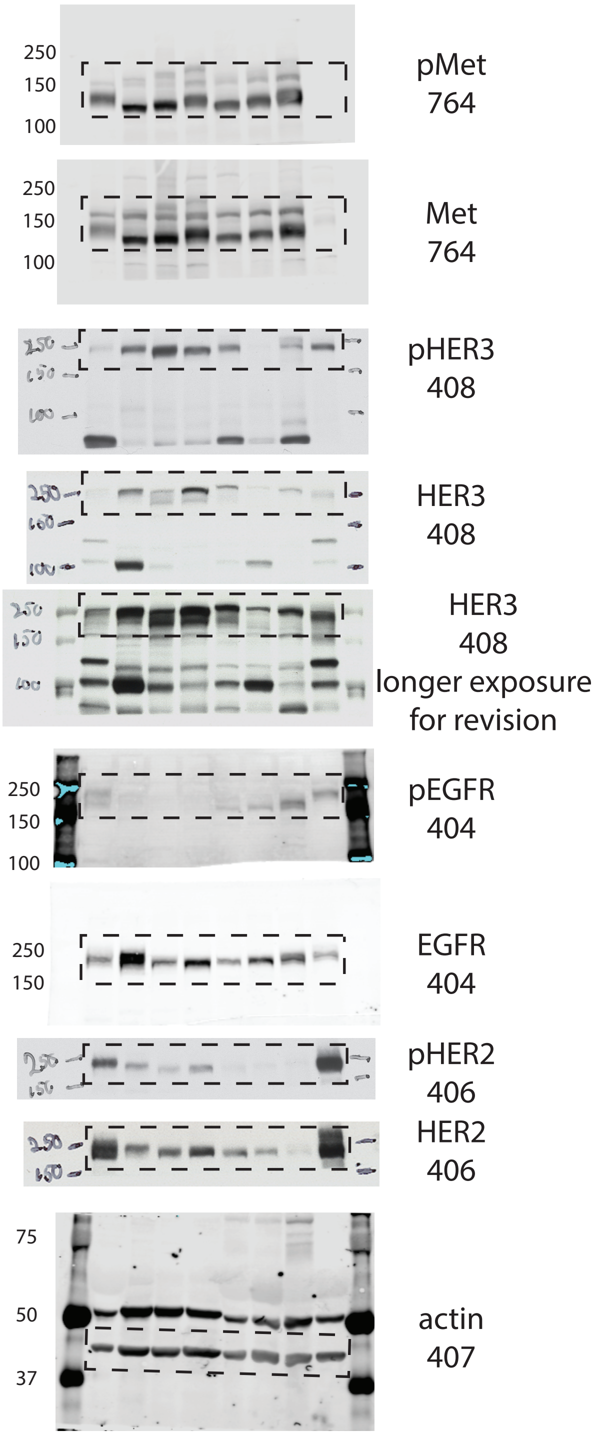
250
150
100
pMet
764
250
150
100
Met
764
pHER3
408
HER3
408
HER3
408
longer exposure
for revision
250
150
100
pEGFR
404
250
150
EGFR
404
pHER2
406
HER2
406
75
50
37
actin
407

Figure 1B

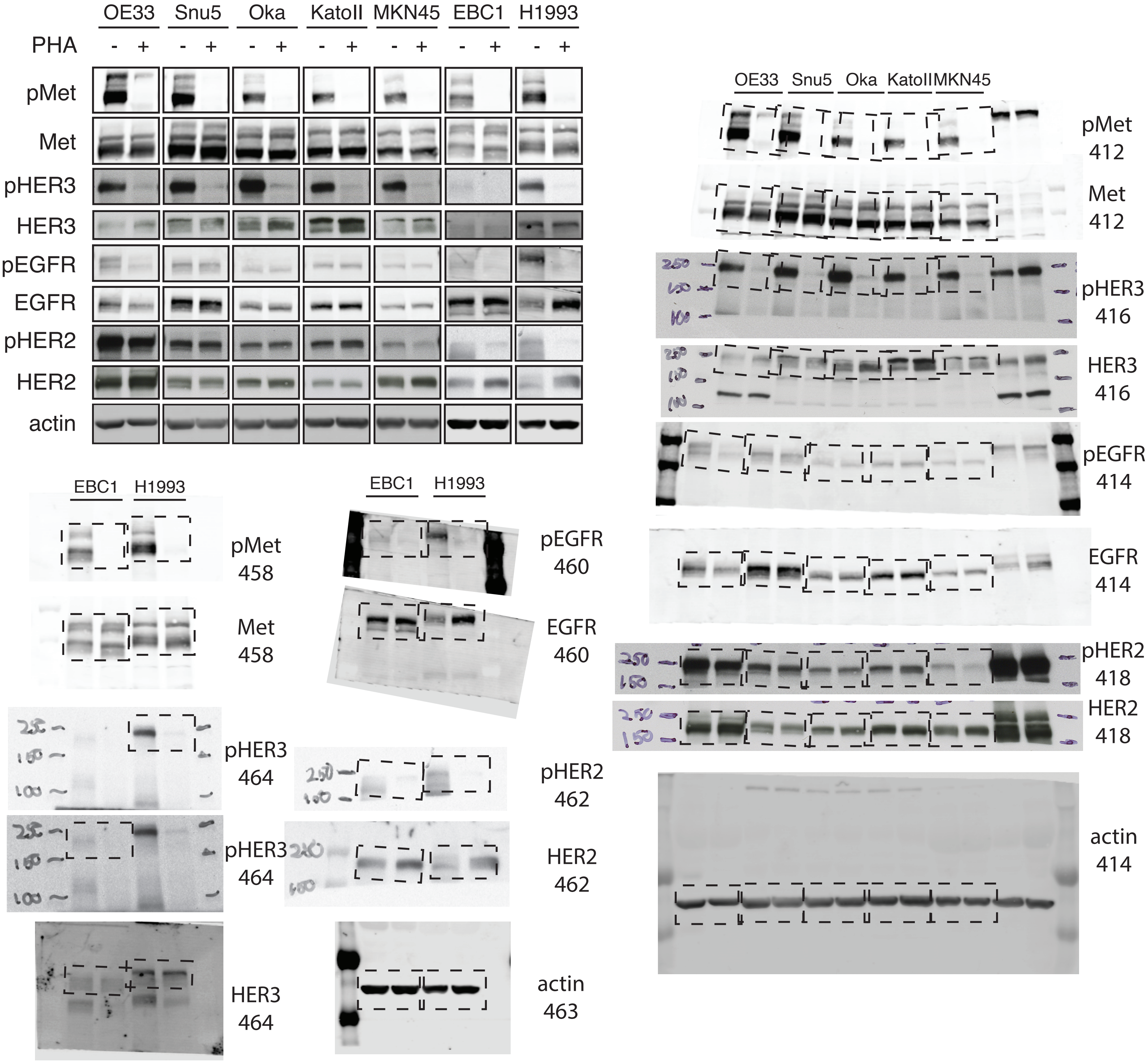
OE33
Snu5
Oka
KatoII
MKN45
EBC1
H1993
PHA
- +
pMet
Met
pHER3
HER3
pEGFR
EGFR
pHER2
HER2
actin
pMet 412
Met 412
pHER3 416
HER3 416
pEGFR 414
EGFR 414
pHER2 418
HER2 418
actin 414
pMet 458
Met 458
pHER3 464
pHER3 464
HER3 464
pEGFR 460
EGFR 460
pHER2 462
HER2 462
actin 463
250
150
100

Figure 1C

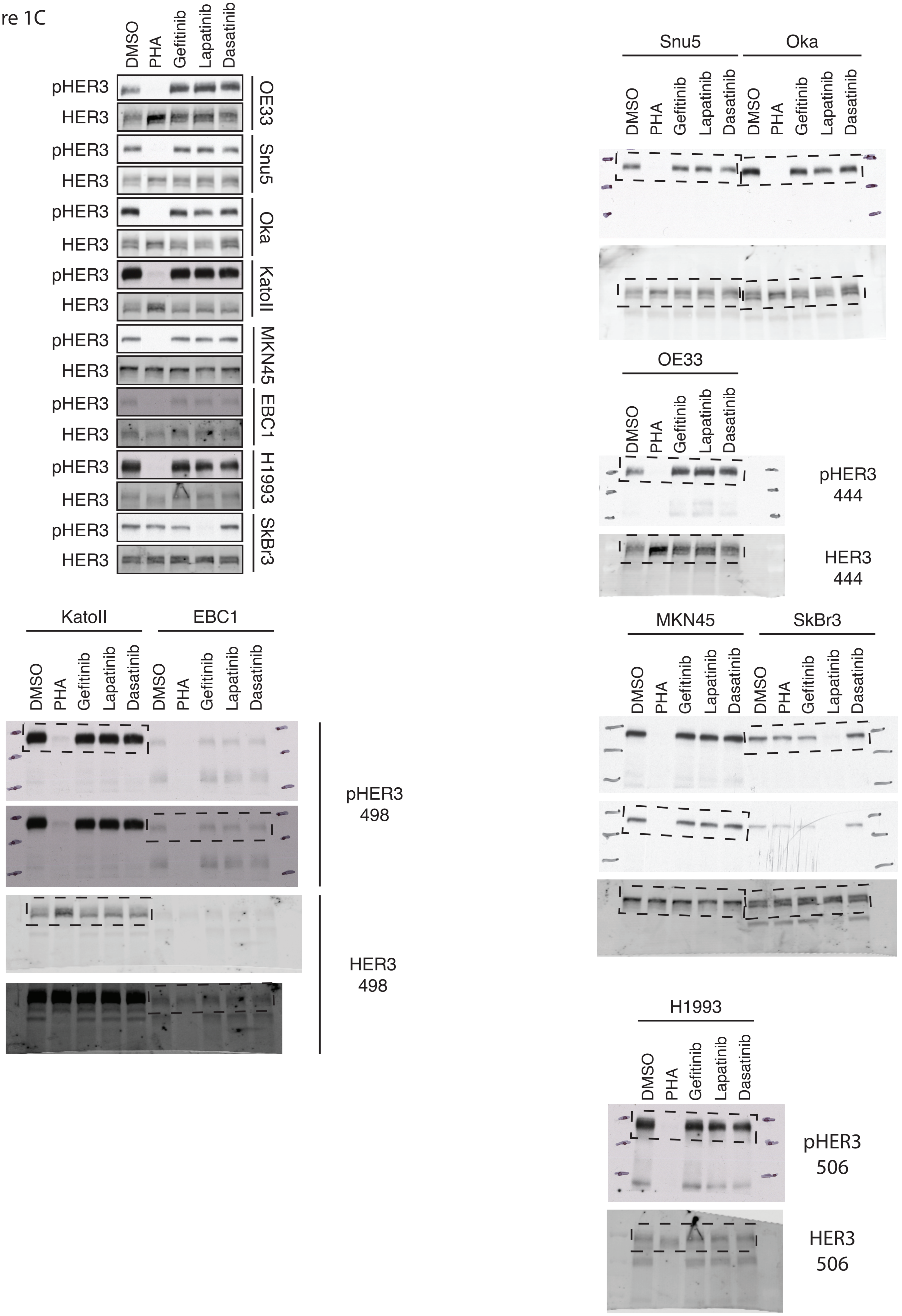
DMSO
PHA
Gefitinib
Lapatinib
Dasatinib
pHER3
HER3
OE33
Snu5
Oka
KatoII
MKN45
EBC1
H1993
SkBr3
pHER3 442
HER3 442
pHER3 444
HER3 444
pHER3 498
HER3 498
pHER3 456
HER3 456
pHER3 506
HER3 506

Figure 2A

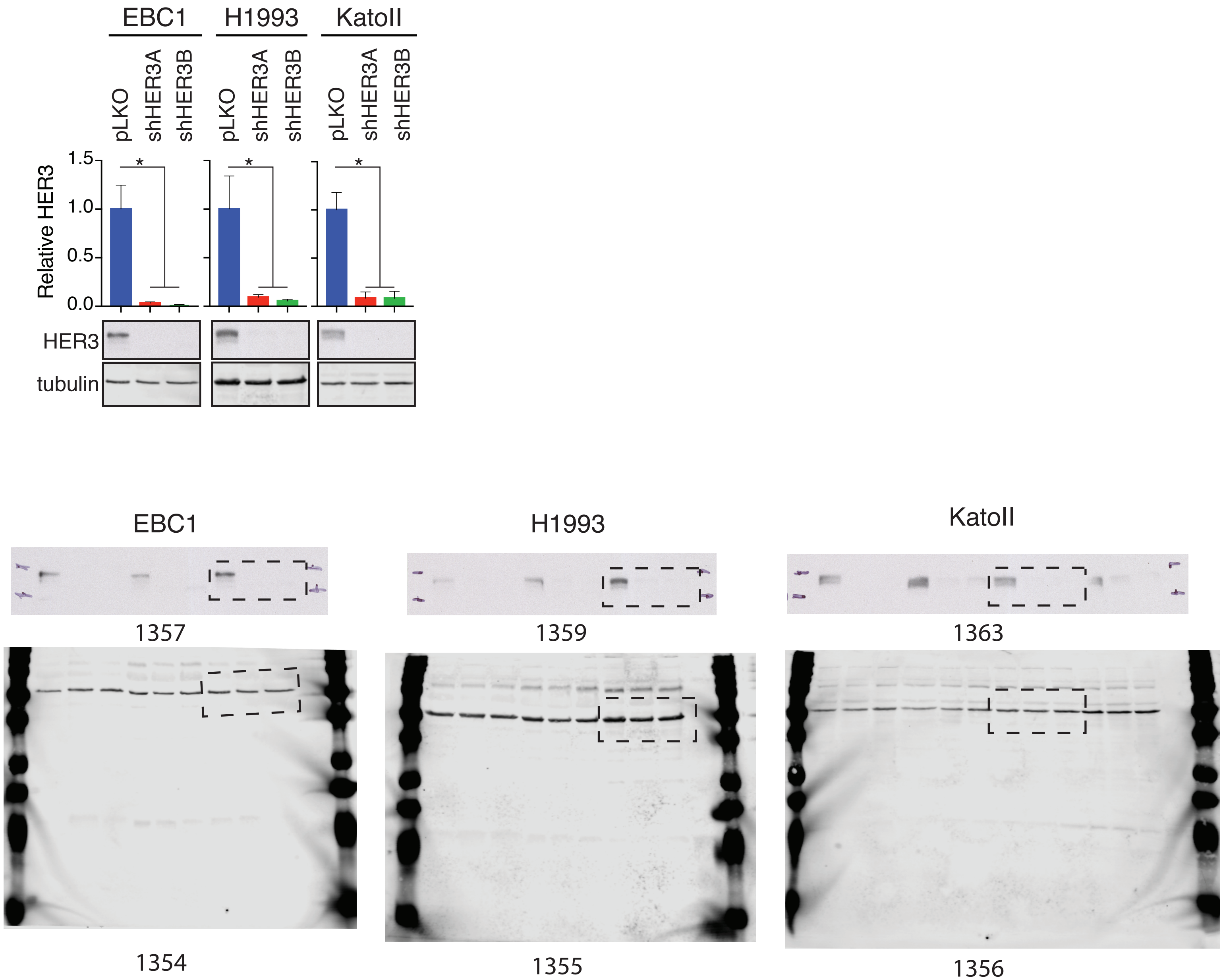
EBC1
H1993
KatoII
pLKO
shHER3A
shHER3B
Relative HER3
1.5
1.0
0.5
0.0
*
HER3
tubulin
EBC1
H1993
KatoII
1357
1359
1363
1354
1355
1356

A

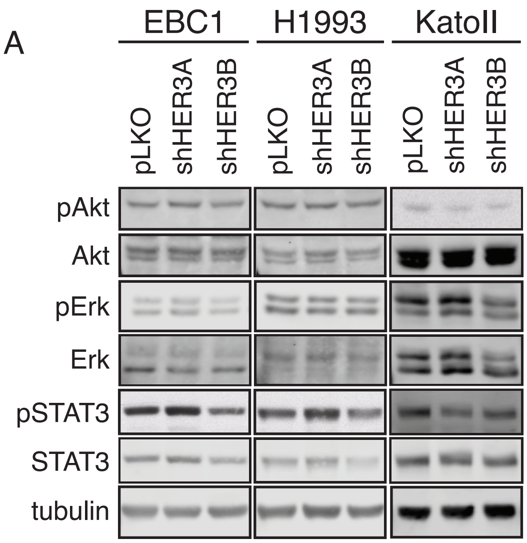

EBC1
H1993
KatoII
pLKO
shHER3A
shHER3B
pAkt
Akt
pErk
Erk
pSTAT3
STAT3
tubulin

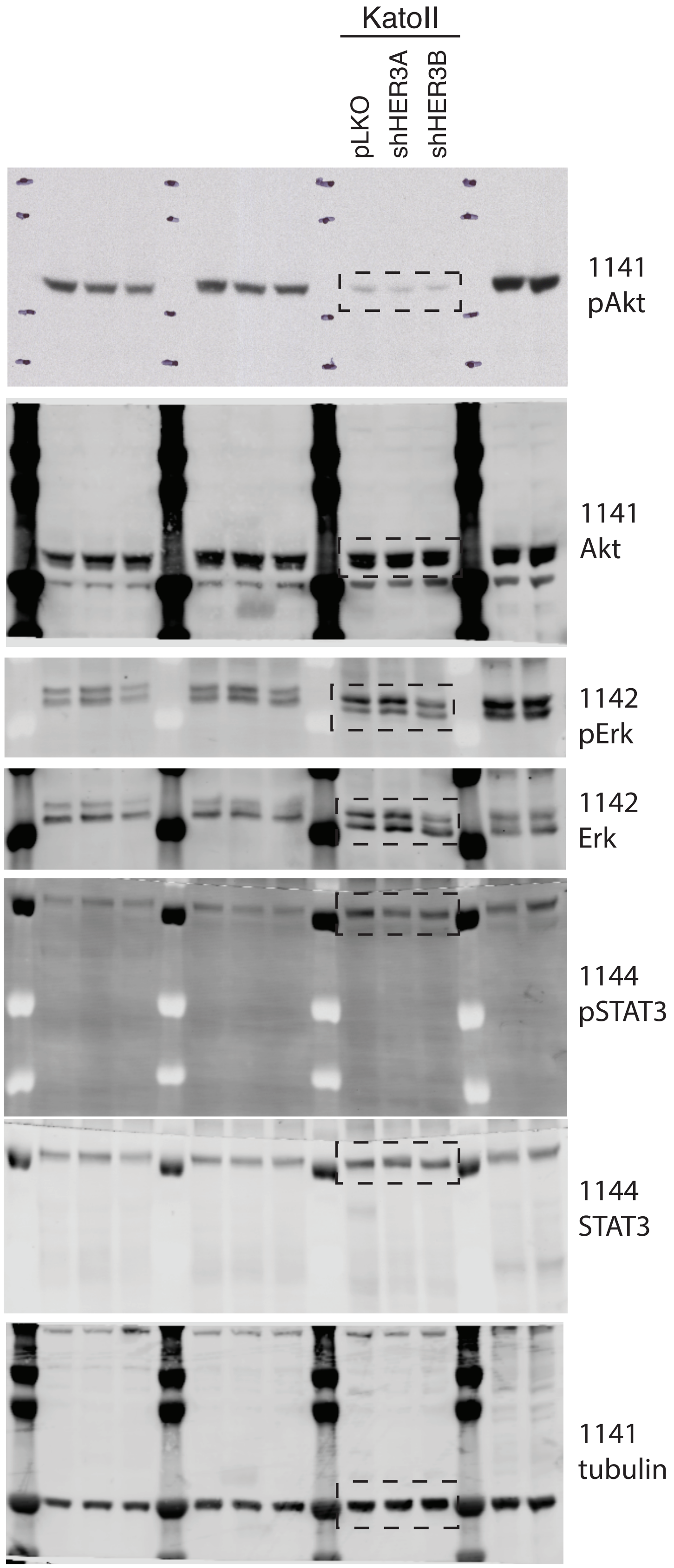

KatoII
pLKO
shHER3A
shHER3B
1141
pAkt
1141
Akt
1142
pErk
1142
Erk
1144
pSTAT3
1144
STAT3
1141
tubulin

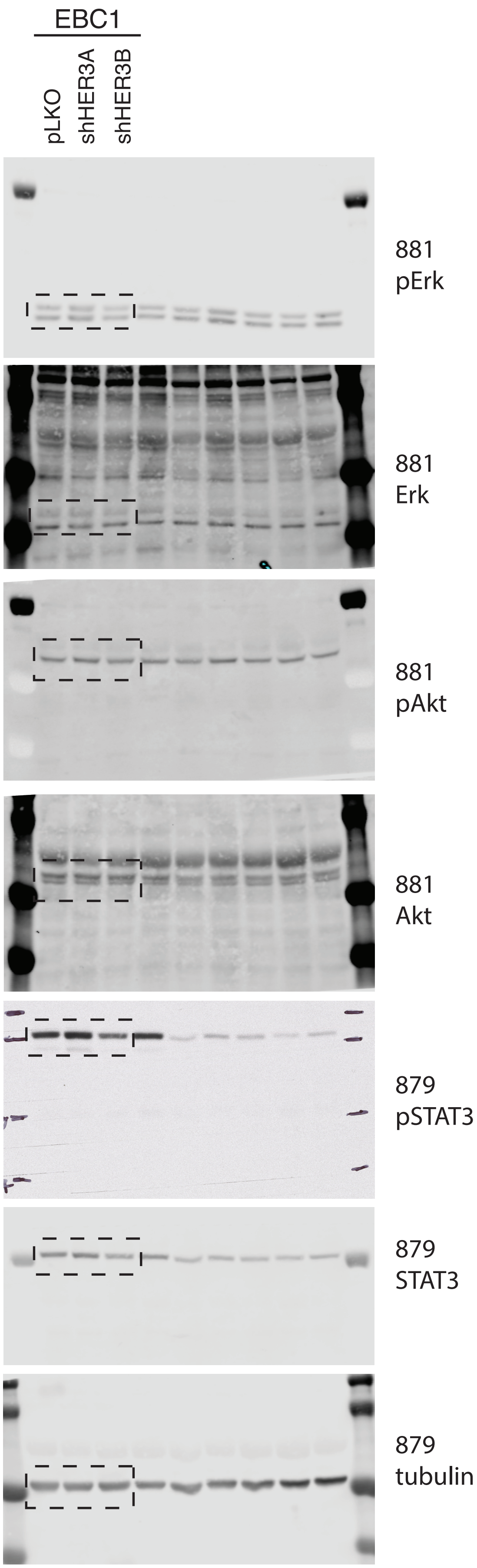
EBC1
pLKO
shHER3A
shHER3B
881
pErk
881
Erk
881
pAkt
881
Akt
879
pSTAT3
879
STAT3
879
tubulin

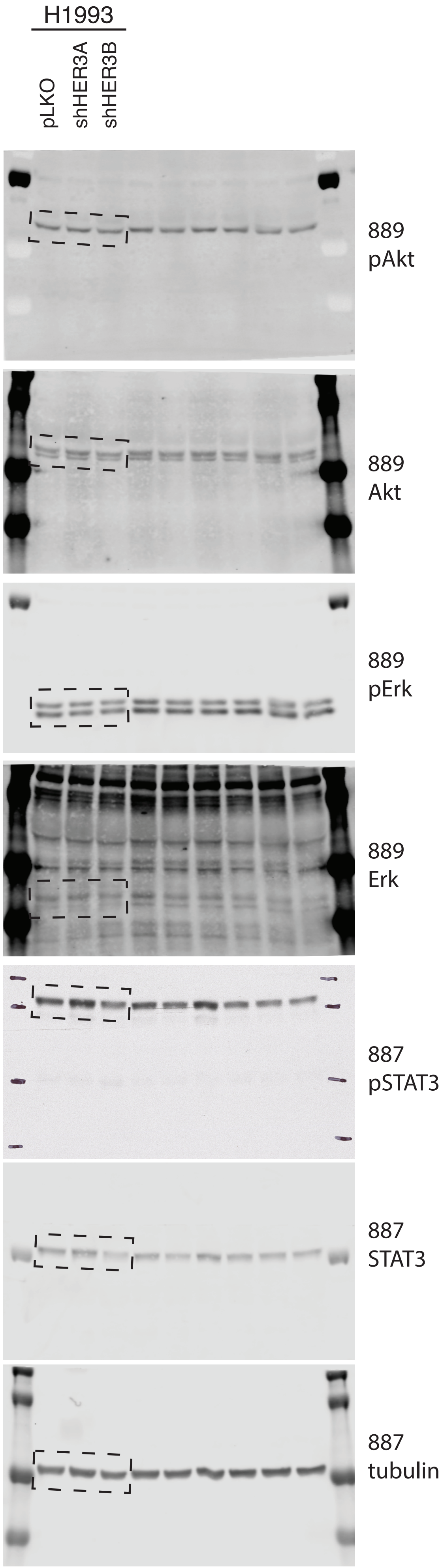
H1993
pLKO
shHER3A
shHER3B
889
pAkt
889
Akt
889
pErk
889
Erk
887
pSTAT3
887
STAT3
887
tubulin

Figure 4A

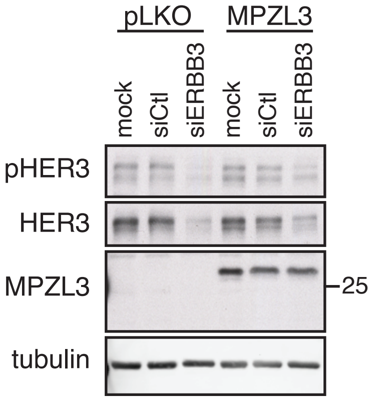

pLKO
MPZL3
mock
siCtl
siERBB3
mock
siCtl
siERBB3
pHER3
HER3
MPZL3
25
tubulin

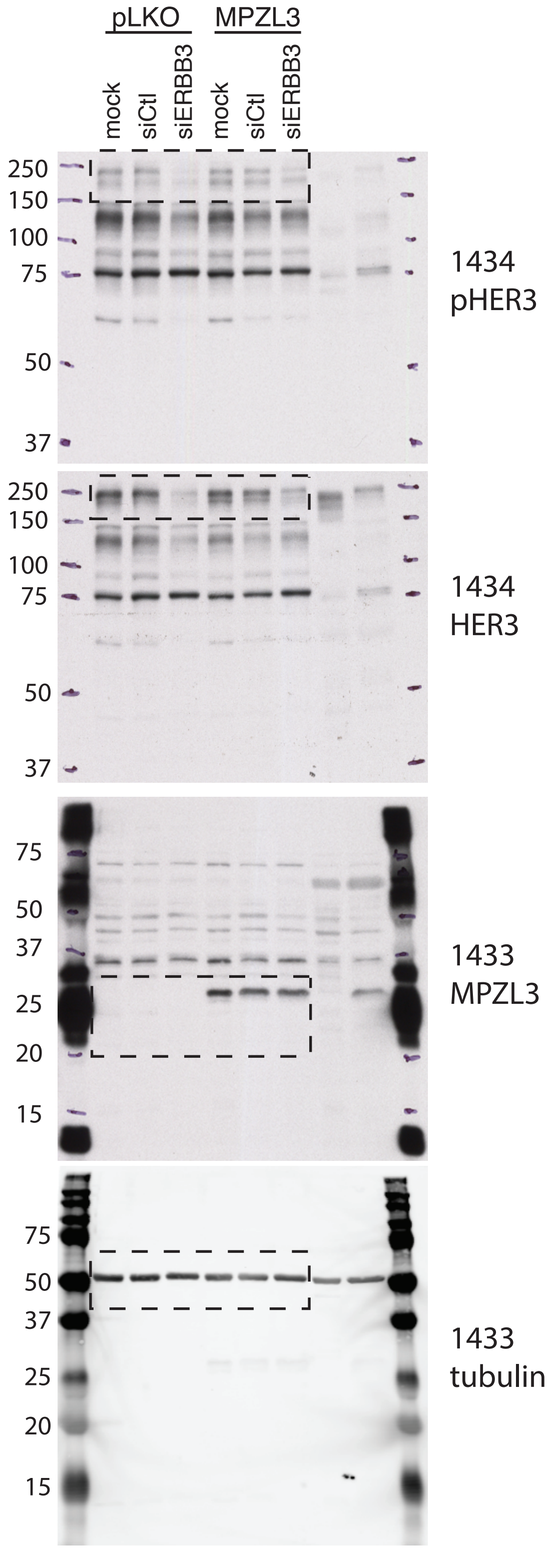

pLKO
MPZL3
mock
siCtl
siERBB3
mock
siCtl
siERBB3
250
150
100
75
50
37
1434
pHER3
250
150
100
75
50
37
1434
HER3
75
50
37
25
20
15
1433
MPZL3
75
50
37
25
20
15
1433
tubulin

Figure 4E

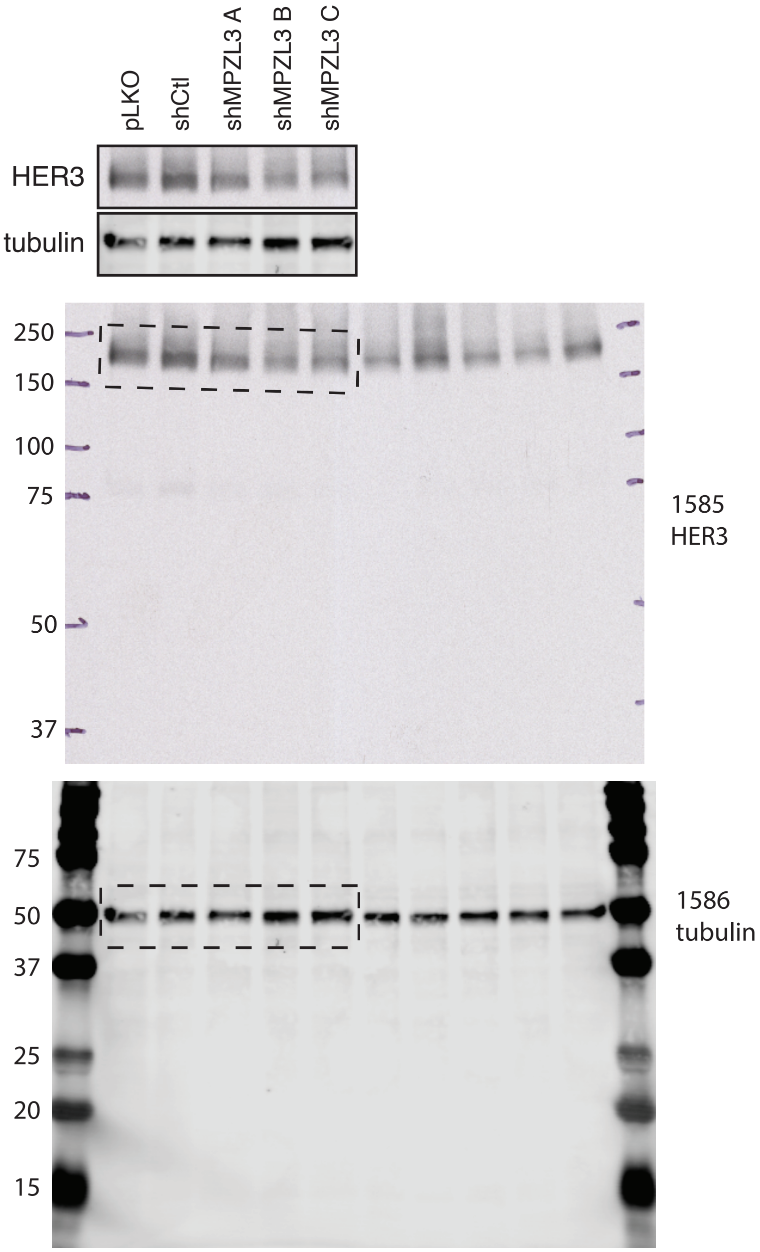

Figure 4H

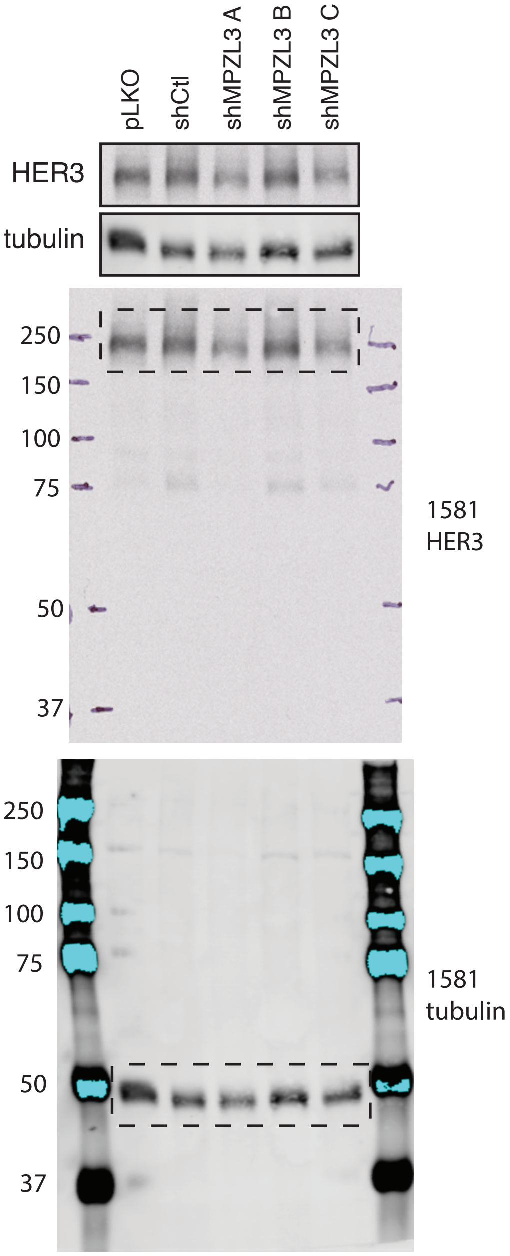

Figure 5A

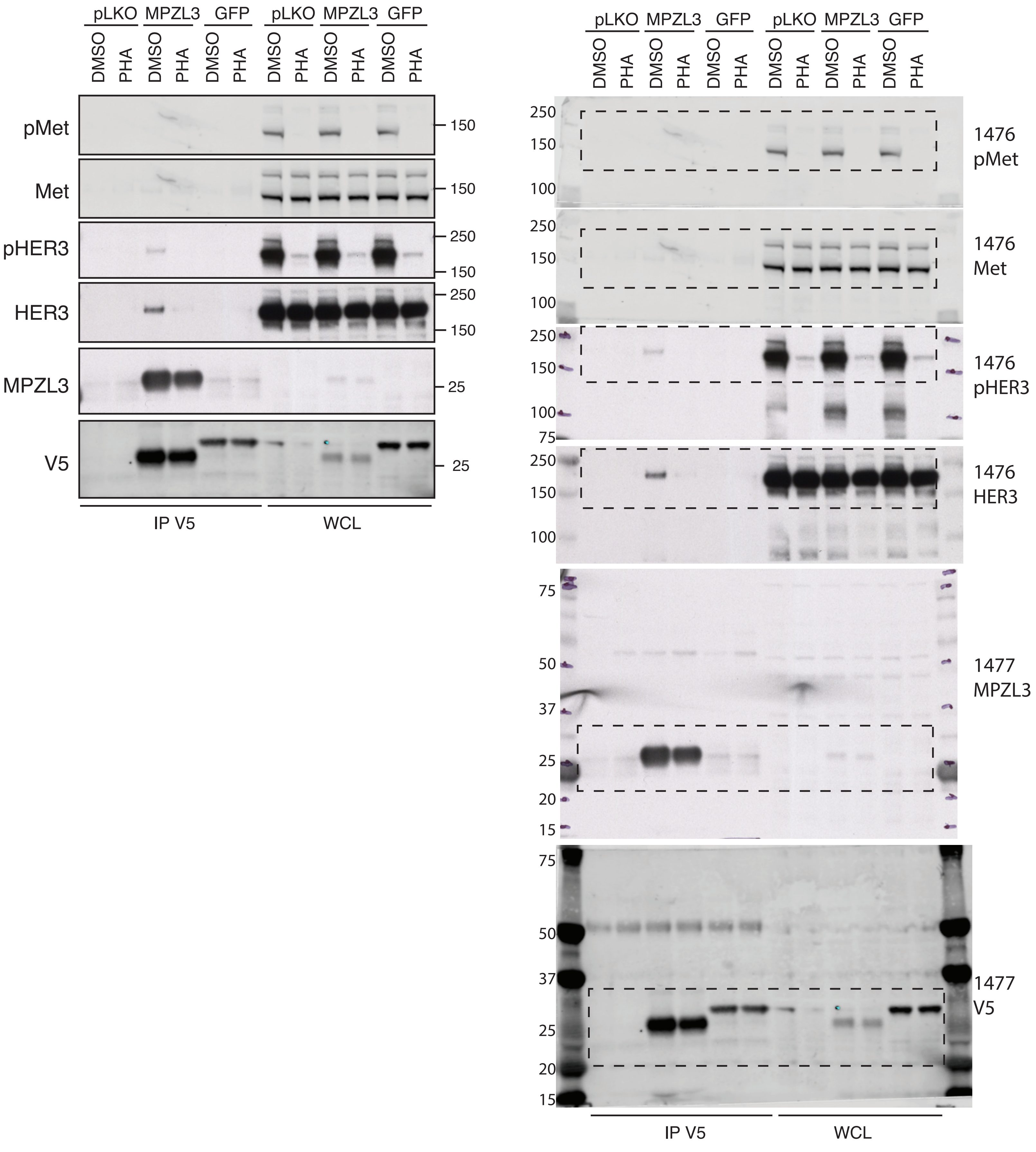
pLKO
MPZL3
GFP
DMSO
PHA
pMet
Met
pHER3
HER3
MPZL3
V5
150
250
25
IP V5
WCL
100
75
50
37
20
15
1476
1477

Figure 5B

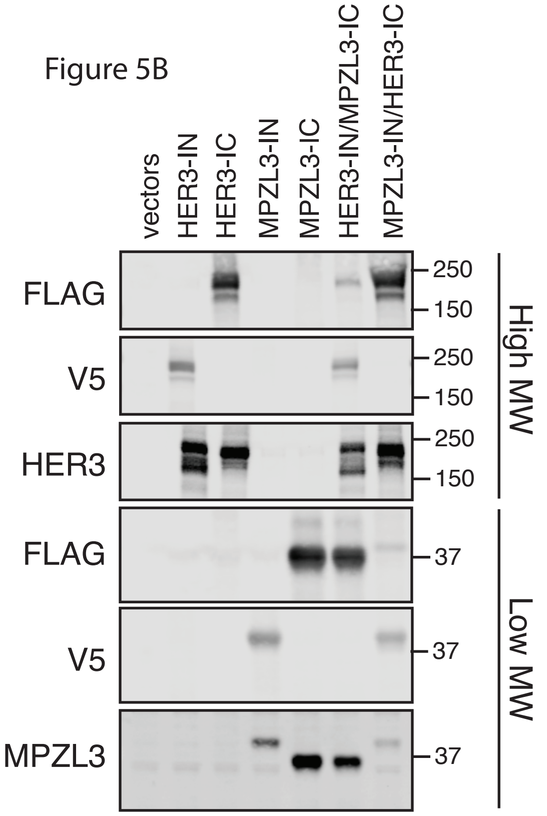
vectors
HER3-IN
HER3-IC
MPZL3-IN
MPZL3-IC
HER3-IN/MPZL3-IC
MPZL3-IN/HER3-IC
FLAG
V5
HER3
FLAG
V5
MPZL3
250
150
250
150
250
150
37
37
37
High MW
Low MW

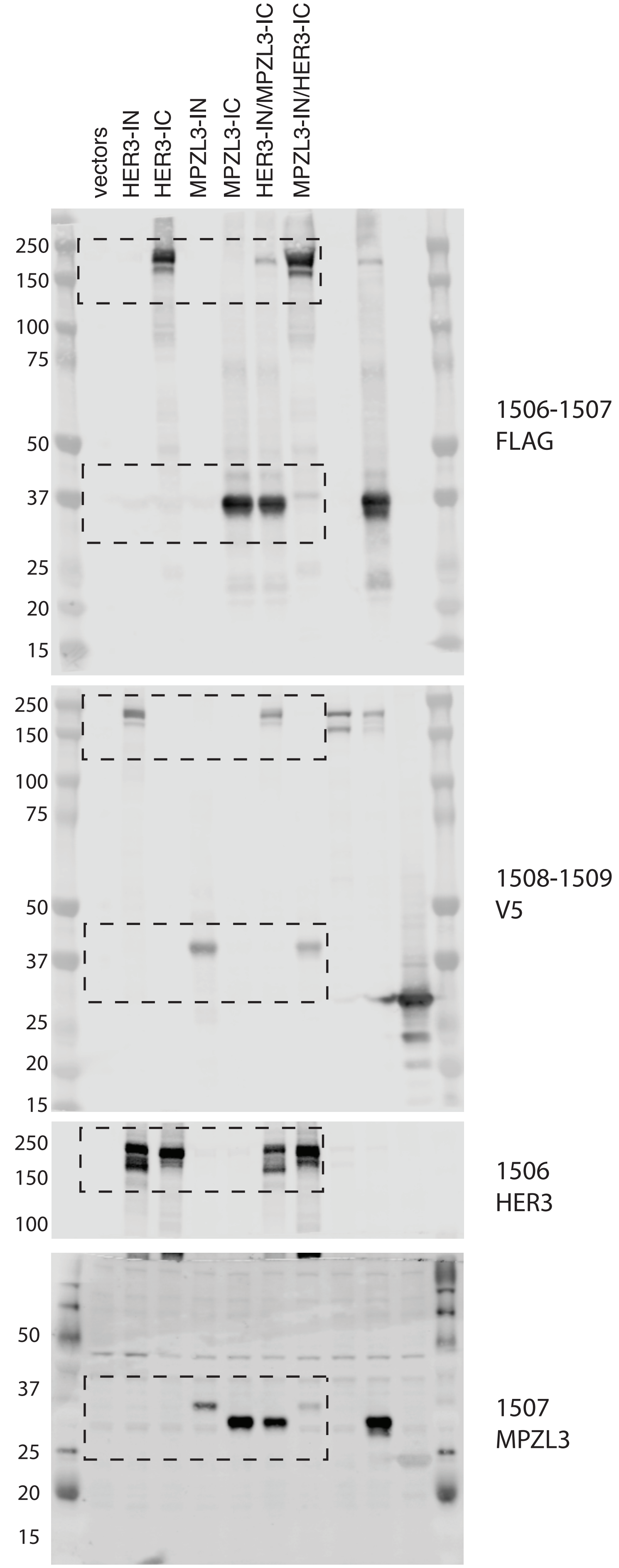
vectors
HER3-IN
HER3-IC
MPZL3-IN
MPZL3-IC
HER3-IN/MPZL3-IC
MPZL3-IN/HER3-IC
250
150
100
75
50
37
25
20
15
1506-1507
FLAG
250
150
100
75
50
37
25
20
15
1508-1509
V5
250
150
100
1506
HER3
50
37
25
20
15
1507
MPZL3

Figure 5D

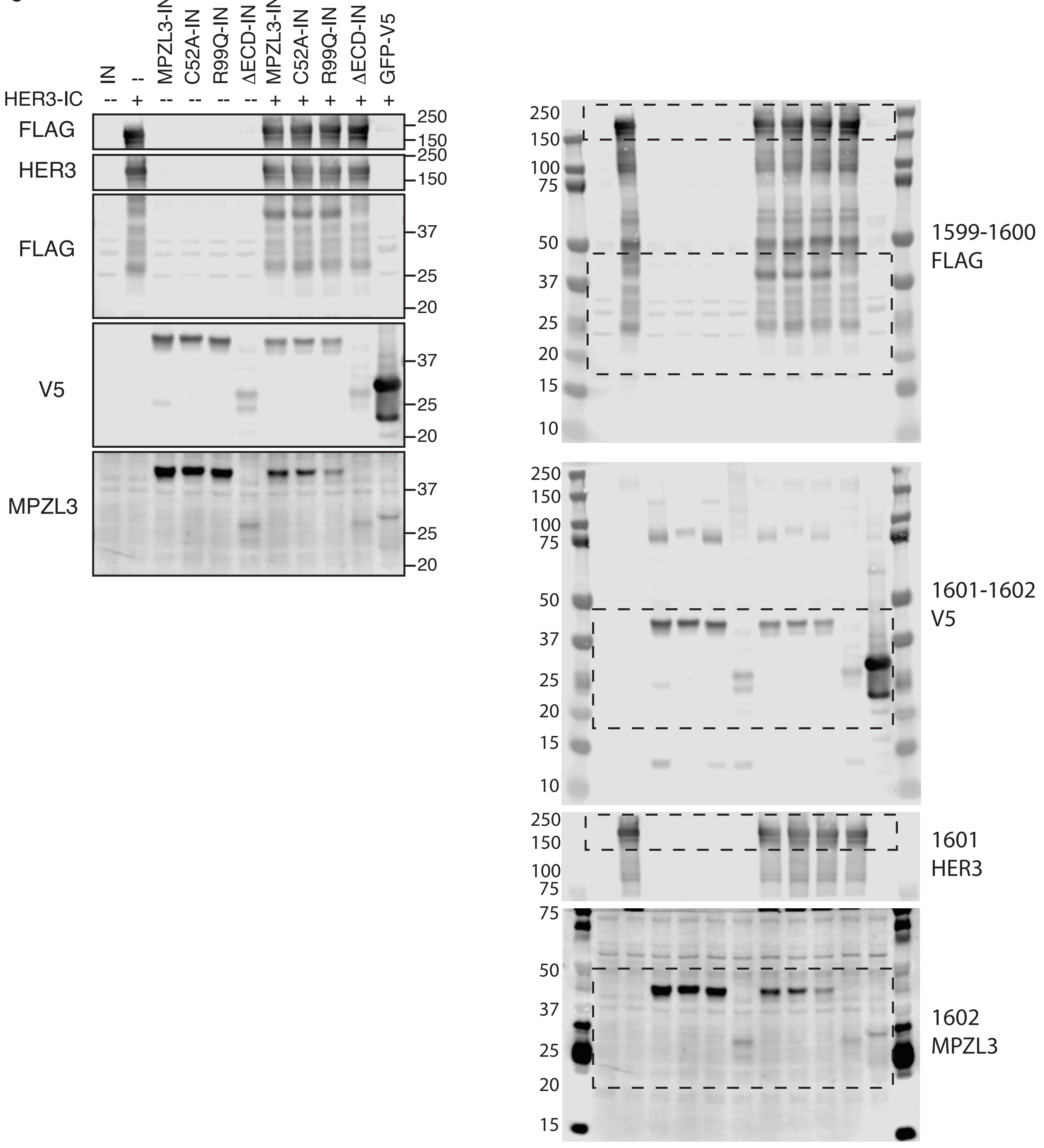
IN
--
MPZL3-IN
C52A-IN
R99Q-IN
ΔECD-IN
MPZL3-IN
C52A-IN
R99Q-IN
ΔECD-IN
GFP-V5
HER3-IC
-- + -- -- -- -- + + + + +
FLAG
HER3
FLAG
V5
MPZL3
250
150
100
75
50
37
25
20
15
10
1599-1600
FLAG
1601-1602
V5
1601
HER3
1602
MPZL3

FigureS1A

A

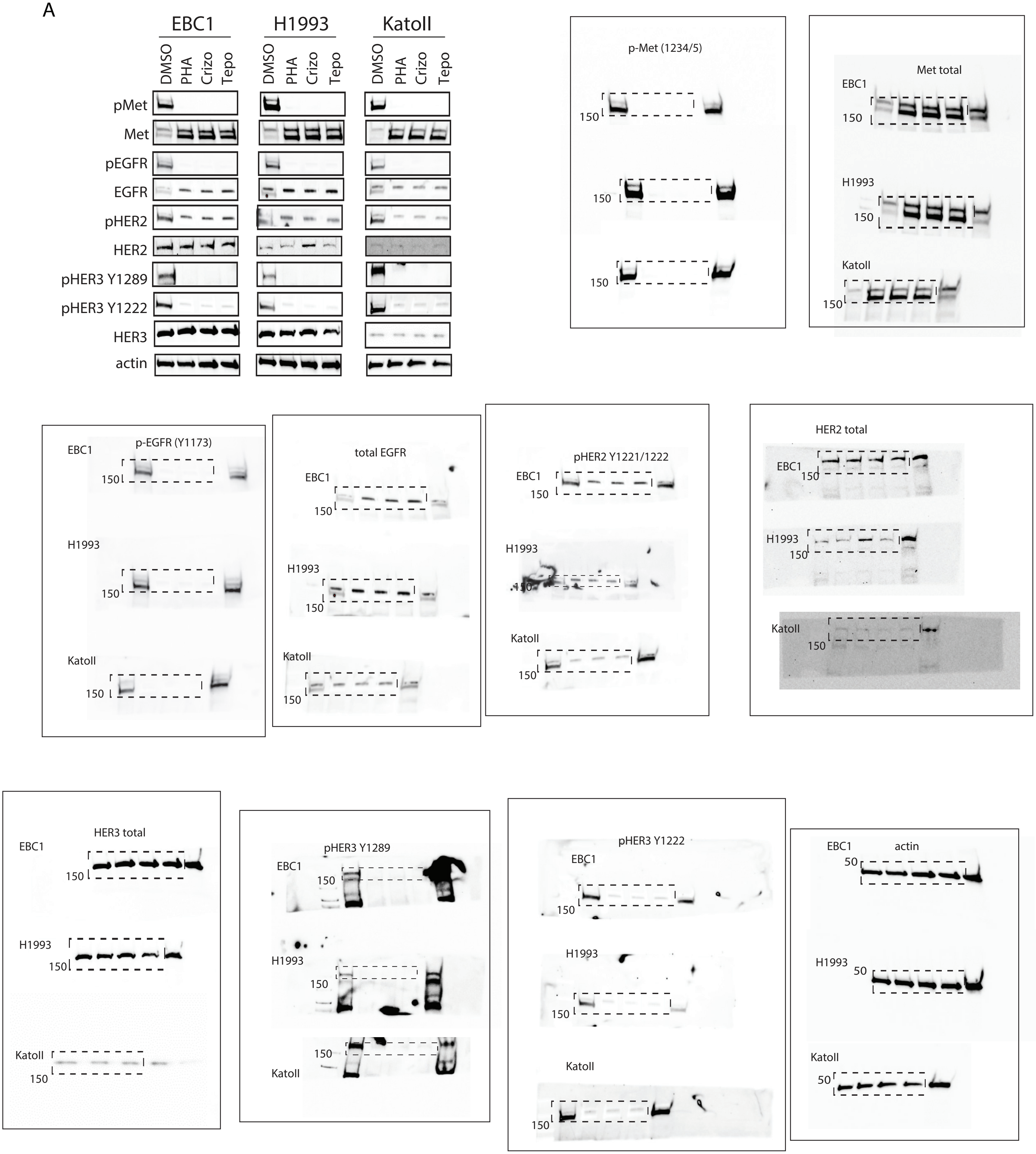
EBC1
H1993
KatoII
DMSO
PHA
Crizo
Tepo
pMet
Met
pEGFR
EGFR
pHER2
HER2
pHER3 Y1289
pHER3 Y1222
HER3
actin
p-Met (1234/5)
150
Met total
p-EGFR (Y1173)
total EGFR
pHER2 Y1221/1222
HER2 total
HER3 total
pHER3 Y1289
pHER3 Y1222
actin
50

Figure S1b

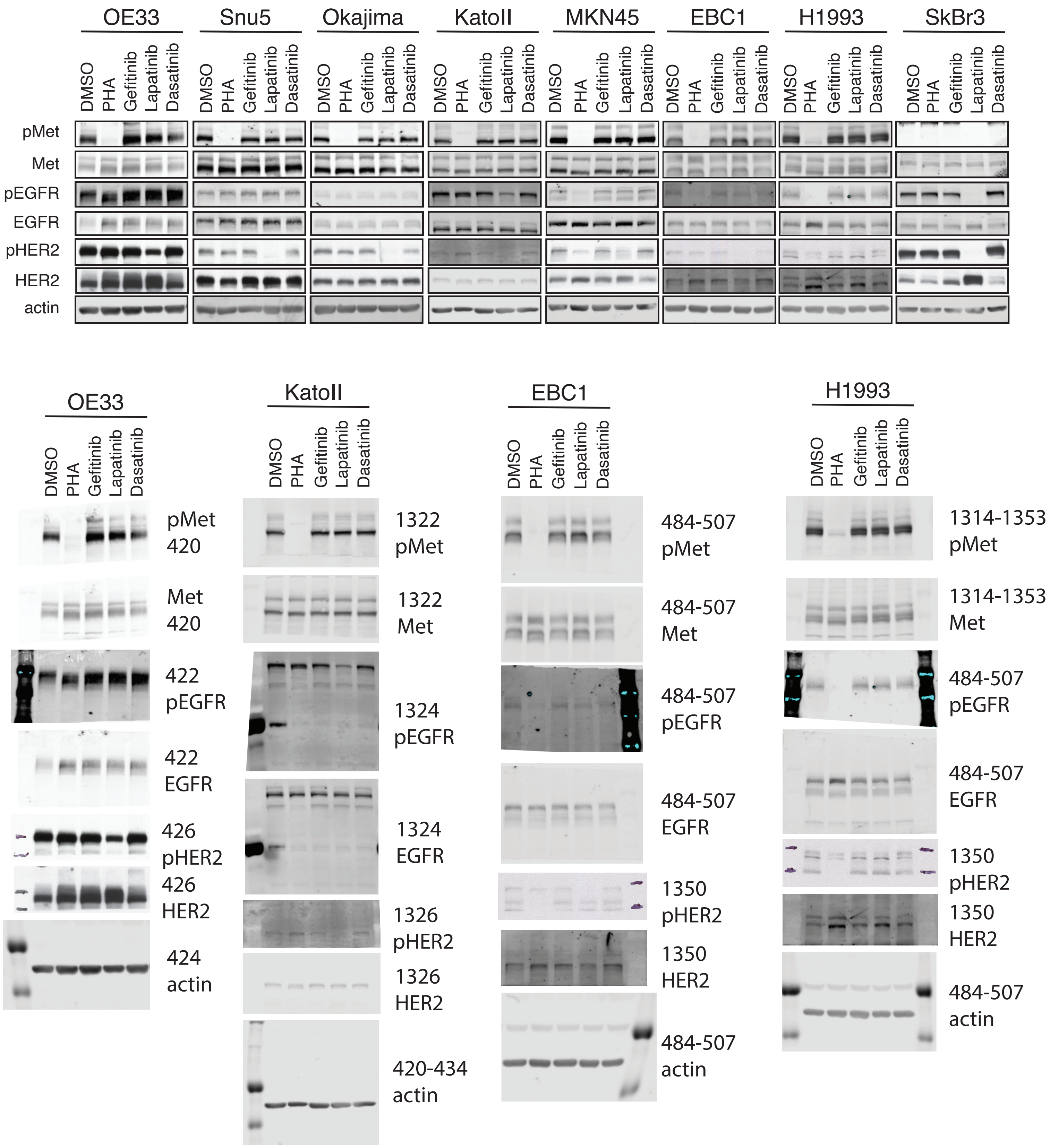
OE33
Snu5
Okajima
KatoII
MKN45
EBC1
H1993
SkBr3
DMSO
PHA
Gefitinib
Lapatinib
Dasatinib
pMet
Met
pEGFR
EGFR
pHER2
HER2
actin
OE33
420 pMet
420 Met
422 pEGFR
422 EGFR
426 pHER2
426 HER2
424 actin
KatoII
1322 pMet
1322 Met
1324 pEGFR
1324 EGFR
1326 pHER2
1326 HER2
420-434 actin
EBC1
484-507 pMet
484-507 Met
484-507 pEGFR
484-507 EGFR
1350 pHER2
1350 HER2
484-507 actin
H1993
1314-1353 pMet
1314-1353 Met
484-507 pEGFR
484-507 EGFR
1350 pHER2
1350 HER2
484-507 actin

Figure S1b

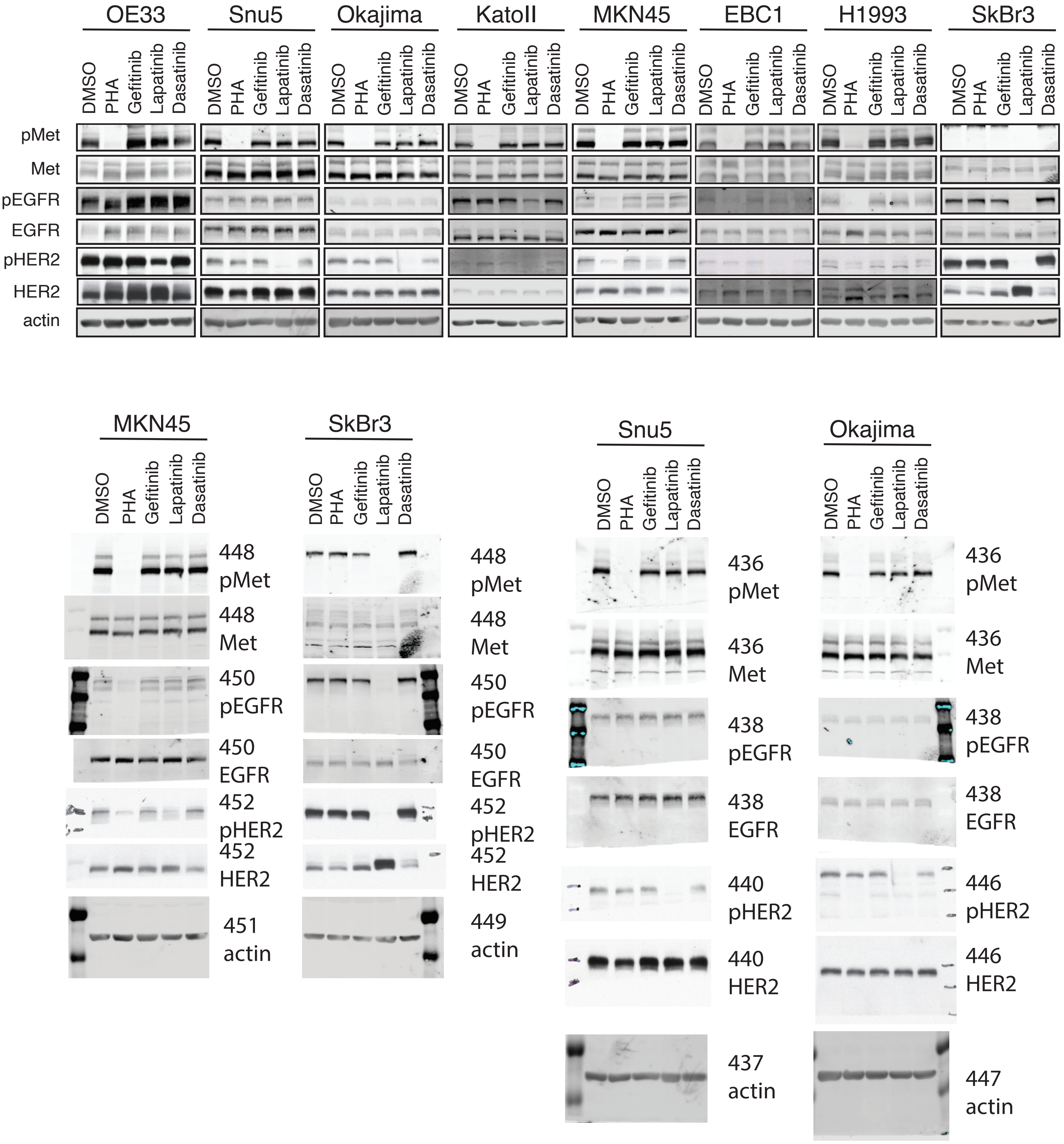
OE33
Snu5
Okajima
KatoII
MKN45
EBC1
H1993
SkBr3
DMSO
PHA
Gefitinib
Lapatinib
Dasatinib
pMet
Met
pEGFR
EGFR
pHER2
HER2
actin
MKN45
SkBr3
Snu5
Okajima
448 pMet
448 Met
450 pEGFR
450 EGFR
452 pHER2
452 HER2
451 actin
448 pMet
448 Met
450 pEGFR
450 EGFR
452 pHER2
452 HER2
449 actin
436 pMet
436 Met
438 pEGFR
438 EGFR
440 pHER2
440 HER2
437 actin
436 pMet
436 Met
438 pEGFR
438 EGFR
446 pHER2
446 HER2
447 actin

Supplementary Figure 1

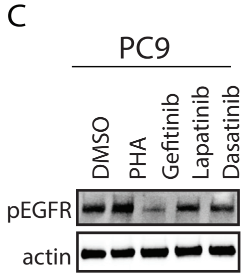

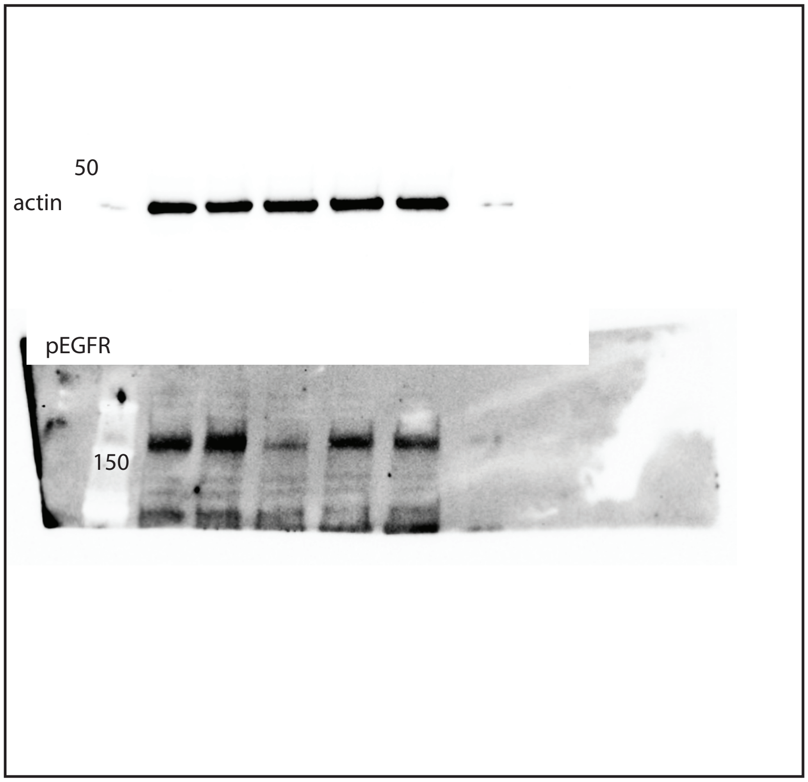

Figure S2

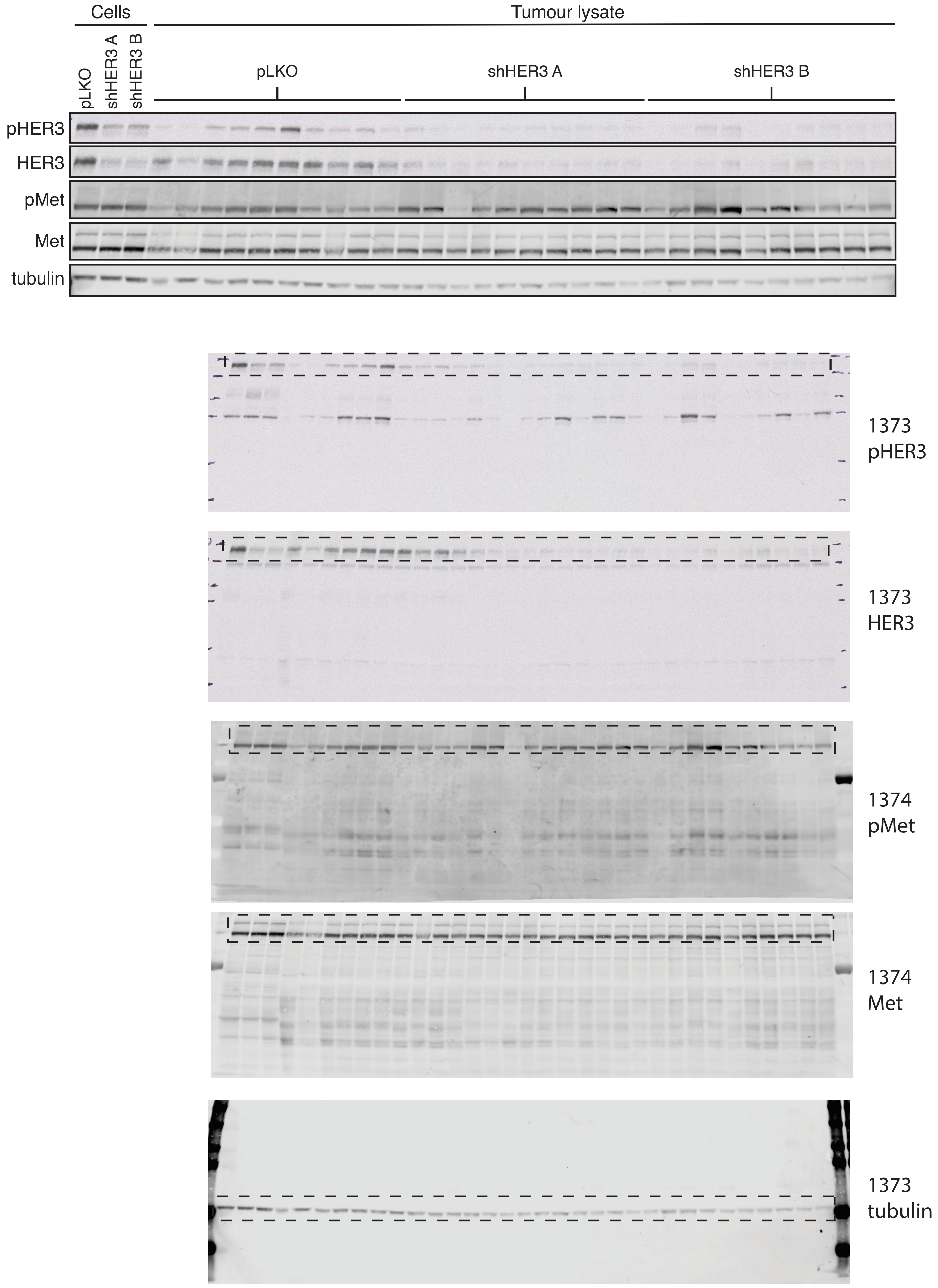
Cells
Tumour lysate
pLKO
shHER3 A
shHER3 B
pLKO
shHER3 A
shHER3 B
pHER3
HER3
pMet
Met
tubulin
1373
pHER3
1373
HER3
1374
pMet
1374
Met
1373
tubulin
